# Custom-made medium approach for effective enrichment and isolation of chemolithotrophic iron-oxidizing bacteria

**DOI:** 10.1093/femsec/fiaf051

**Published:** 2025-05-05

**Authors:** Tomoki Uchijima, Shingo Kato, Kazuya Tanimoto, Fumito Shiraishi, Natsuko Hamamura, Kohei Tokunaga, Hiroko Makita, Momoko Kondo, Moriya Ohkuma, Satoshi Mitsunobu

**Affiliations:** Department of Science and Technology for Biological Resources and Environment, Graduate School of Agriculture, Ehime University, 3-5-7 Tarumi, Matsuyama, Ehime 790-8566, Japan; Japan Collection of Microorganisms, RIKEN BioResource Research Center, 3-1-1 Koyadai, Tsukuba, Ibaraki 305-0074, Japan; Department of Science and Technology for Biological Resources and Environment, Graduate School of Agriculture, Ehime University, 3-5-7 Tarumi, Matsuyama, Ehime 790-8566, Japan; Earth and Planetary Systems Science Program, Graduate School of Advanced Science and Engineering, Hiroshima University, 1-3-1 Kagamiyama, Higashi-Hiroshima, Hiroshima 739-8526, Japan; Department of Biology, Graduate School of Science, Kyushu University, 744 Motooka, Nishi-ku, Fukuoka 819-0395, Japan; Ningyo-Toge Environmental Engineering Center, Japan Atomic Energy Agency, Okayama 708-0698, Japan; Department of Ocean Sciences, Tokyo University of Marine Science and Technology, 4-5-7 Konan, Minato-ku, Tokyo 108-8477, Japan; Institute for Extra-Cutting-Edge Science and Technology Avant-Garde Research, (X-star), Super-cutting-edge Grand and Advanced Research (SUGAR) Program, Japan Agency for Marine-Earth Science and Technology (JAMSTEC), Yokosuka 237-0061, Japan; Department of Science and Technology for Biological Resources and Environment, Graduate School of Agriculture, Ehime University, 3-5-7 Tarumi, Matsuyama, Ehime 790-8566, Japan; Japan Collection of Microorganisms, RIKEN BioResource Research Center, 3-1-1 Koyadai, Tsukuba, Ibaraki 305-0074, Japan; Department of Science and Technology for Biological Resources and Environment, Graduate School of Agriculture, Ehime University, 3-5-7 Tarumi, Matsuyama, Ehime 790-8566, Japan

**Keywords:** custom-made medium, enrichment, Gallionellaceae, iron-oxidizing bacteria, isolation

## Abstract

Chemolithotrophic neutrophilic iron (Fe)-oxidizing bacteria, which mainly belong to the family Gallionellaceae, universally prevail in terrestrial environments changing Fe cycling. However, they are typically recognized as difficult-to-culture microbes. Despite efforts, there are few Fe(II)-oxidizing lithotroph isolates; hence, their physiological and ecological knowledge remains limited. This limitation is largely owing to difficulties in their cultivation, and we hypothesize that the difficulty exists because substrate and mineral concentrations in the cultivation medium are not tuned to a specific environmental condition under which these organisms live. To address this hypothesis, this study proposes a novel custom-made medium approach for chemolithotrophic Fe(II)-oxidizing bacteria; a method that manipulates medium components through diligent analysis of field environment. A new custom-made medium simulating energy substrates and nutrients under the field condition was prepared by modifying both chemical composition and physical setup in the glass-tube medium. In particular, the modification of the physical setup in the tube had a significant effect on adjusting dissolved Fe(II) and O_2_ concentrations to the field environment. Using the medium, Gallionellaceae members were successfully enriched and a new *Gallionellaceae* species was isolated from a natural hot spring site. Compared with conventional medium, the custom-made medium has significantly higher ability in enriching Gallionellaceae members.

## Teaser

“Custom-made” medium approach to effectively enrich and isolate the difficult-to-culture chemolithotrophic iron-oxidizing bacteria from the natural environment.

## Introduction

On Earth, chemolithotrophic iron (Fe) oxidizing bacteria thrive by catalyzing the exergonic ferrous iron [Fe(II)] oxidation with O_2_ (Roden et al. 2004, Emerson et al. 2010, Melton et al. 2014).


\begin{eqnarray*}
{\mathrm{F}}{{\mathrm{e}}^{2 + }} + 2.5{{\mathrm{H}}_2}{\mathrm{O}} + 0.25{{\mathrm{O}}_2} \to {\mathrm{Fe}}{\left( {{\mathrm{OH}}} \right)_3} \downarrow + 2{{\mathrm{H}}^ + }
\end{eqnarray*}

In particular, a circumneutral pH environment prevails at the Earth’s surface, and neutrophilic terrestrial Fe(II)-oxidizing lithotrophs, most of which known so far belong to the family Gallionellaceae, are universally found in various Fe(II)-rich environments (e.g. groundwater, marshes, hot springs, mine drainages, wetland plant rhizospheres), thereby changing the Fe cycling (Emerson and Moyer 1997, Bruneel et al. 2006, Weiss et al. 2007, Emerson et al. 2010, Krepski et al. 2012, Mitsunobu et al. 2012, Fabisch et al. 2013, Kato et al. 2013, Chan et al. 2023). Neutrophilic Fe(II)-oxidizing bacteria certainly form the poorly crystalline Fe(III) oxyhydroxides with high sorption capacity (Fortin and Langley 2005, Chan et al. 2009, Miot et al. 2009, Mitsunobu et al. 2012) due to very low solubility of Fe(III) at a neutral pH (Cornell and Schwertmann 2003). This consequently affects the behaviors of other important elements (e.g. P, As, U, Cu, Se, Cs) at the Earth’s surface (Ferris et al. 2000, Katsoyiannis and Zouboulis 2004, Katsoyiannis et al. 2006, Fabisch et al. 2013, Mitsunobu et al. 2013, Sowers et al. 2017, Field et al. 2019, Kikuchi et al. 2019). In addition, in the neutral pH range, microbial Fe(II) oxidation rates are comparable to the chemical oxidation rates (Singer and Stumm 1970, Roden et al. 2004). Interestingly, these bacteria favor a low-O_2_ environment with relatively slow chemical oxidation to avoid competition (Neubauer et al. 2002, Emerson et al. 2010). The dynamic competition renders cultivation of neutrophilic Fe(II)-oxidizing bacteria in the laboratory challenging (Emerson and Moyer 1997, Summers et al. 2013, Lueder et al. 2022, Zhou et al. 2022); hence these bacteria are recognized as difficult-to-culture microbes (Summers et al. 2013, Lueder et al. 2022, Zhou et al. 2022).

Most cultivation techniques for neutrophilic Fe(II)-oxidizing bacteria are traced to a method developed by Kucera and Wolfe (1957) ~70 years ago. They developed a simple but effective culture forming opposite gradients of O_2_ and Fe(II) by adding a solid Fe(II) source to a tube containing a mineral salt medium. Hanert (1981) made an important compositional modification of the mineral medium, and called it the Modified Wolfe’s Mineral Medium (MWMM). Emerson and Moyer (1997) further modified the medium by adding a low concentration agarose, which allowed unicellular Fe(II)-oxidizing bacteria to remain stable and grow at their optimum Fe(II) and O_2_ levels. Currently, the agarose-stabilized gradient tube with MWMM and a solid Fe(II) source is a standard medium for cultivating terrestrial neutrophilic Fe(II)-oxidizing lithotrophs (Naik and Patel 2022), which is also possible under microoxic conditions in Petri dishes containing solid Fe(II) (Emerson and Weiss 2004) or in a liquid medium with dissolved Fe(II) and micromolar O_2_ (Maisch et al. 2019).

Although many researchers have attempted to enrich and isolate the neutrophilic chemolithotrophic Fe(II)-oxidizing bacteria using these media, the number of isolates has been relatively low. In some cases, the enrichment of chemolithotrophic Fe(II)-oxidizers was unsuccessful using the gradient medium with MWMM (Wang et al. 2009, Hassan et al. 2016, Tong et al. 2019). Also, it is often difficult to keep them alive after/in multiple serial dilutions and transfers as with other autotrophs (Gupta and Agate 1986, Tokuyama 1994). Currently, there are seven *Gallionellaceae* isolates and several stable enrichment cultures (Emerson and Moyer 1997, Lüdecke et al. 2010, Krepski et al. 2012, Kato et al. 2014, Khalifa et al. 2018, Kato et al. 2022, Hoover et al. 2023). However, cultivation-independent phylogenetic analysis has identified the presence of numerous unclassified and uncultured *Gallionellaceae* in the environment (Liljeqvist et al. 2015, Parks et al. 2017, Jakus et al. 2021, Chan et al. 2023), indicating that the ecological and physiological knowledge of these organisms remains limited. This limitation is largely owing to difficulties in the cultivation (Summers et al. 2013, Lueder et al. 2022, Zhou et al. 2022). We hypothesize that this difficulty exists because the substrate and mineral concentrations in the medium are not properly tuned to the field conditions under which the Fe(II)-oxidizers live. The concentrations of iron, oxygen, major elements, and carbonate ions in terrestrial water are very diverse (Langmuir 1997, Stumm and Morgan 2012). Consequently, the total ion abundance also varies widely across different terrestrial environments, with reports of salinity ranging from very low to very high, similar to that of seawater (Langmuir 1997, Appelo and Postma 2005). Also, in the most of neutral terrestrial water, the pH values are strongly regulated by the dissolved carbonate ions (Stumm and Morgan 2012). Hence, the carbonate abundance is a key factor in the medium composition for autotrophic Fe(II)-oxidizing bacteria, both in terms of carbon source and pH regulation. From these perspectives, the one type of medium MWMM designated for a deep-well water (Kucera and Wolfe 1957, Hanert 1981 , Hanert 2006) cannot represent the diverse freshwater environments, and thus adjusting the medium composition would be a better alternative. However, the MWMM with Fe(II) source, has been predominantly used for their enrichments and isolations (Emerson and Moyer 1997, Weiss et al. 2007, Wang et al. 2009, Lüdecke et al. 2010, Krepski et al. 2012, Kato et al. 2013, Hassan et al. 2016, Khalifa et al. 2018, Tong et al. 2019, Tong et al. 2021 , Lueder et al. 2022). To address this issue, this study proposed a new custom-made medium approach for chemolithotrophic Fe(II)-oxidizing bacteria that manipulates the medium composition based on physicochemical analysis of the field environment. The concentrations of dissolved Fe(II), O_2_, and primary nutrient elements were adjusted to the field condition in the custom-made medium. In particular, changing the physical setup in the glass-tube medium (placements of solid Fe(II) source and growth zone) assisted in adjusting the Fe(II) and O_2_ concentrations to the field levels and providing a suitable habitat for the Fe(II)-oxidizing bacteria in the medium. Using this approach, new isolates were obtained from a natural geothermal spring.

## Materials and methods

### Site description and inoculation source

**Figure 1.**
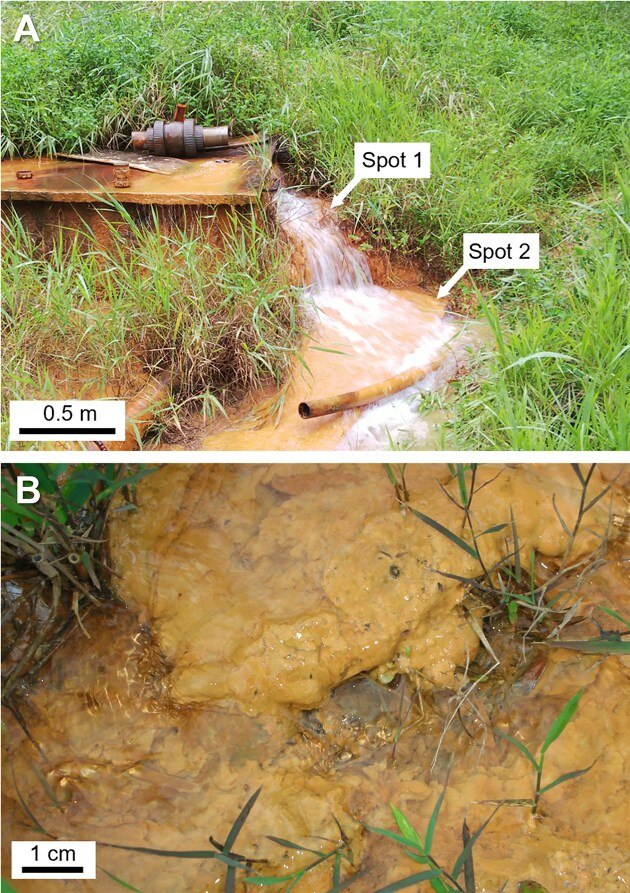
(A) Sampling location in the Sambe hot spring site and (B) Fe precipitates with a soft structure at the spot 2.

The field site was an Fe-enriched hot spring in the Sambe geothermal area (35.12°N, 132.63°E) in Shimane, Japan. This hot spring is located at the foot of Mt. Sambe, an active volcano (Mitsunobu et al. 2012). The moderately warm spring water (ca. 30°C) gushes out from several rock cracks, and yellowish Fe oxide precipitates cover the floor and exist in the stream along the flow from the spring source (Fig. 1A). The Fe oxide precipitates collected at the Sambe hot spring site were used for the inoculation source for the target Fe(II)-oxidizing bacteria. The Fe oxide precipitates were loosely aggregated with a soft structure (Fig. 1B) and carefully sampled in a sterile 50-ml plastic tube with a sterile spatula, immediately sealed, kept cold (ca. 5°C) during transportation to our laboratory, and stored at 4°C in a dark refrigerator prior to use.

### Chemical and mineralogical analyses

The chemical composition and physical condition of spring water are summarized in Table 1. The data in Table 1 are based on two field trips performed in February and December 2022. Spring water temperatures, temperature-compensated pH, and dissolved O_2_ (DO) concentrations were measured at the site using a portable pH meter (D-51, Horiba) with a glass electrode (Horiba, 9625–10D) and a DO meter (HQ30d, Hach Toa-Dkk) by directly inserting the electrodes into the spring water. The electrical conductivity (EC) was measured using a portable EC meter (D-210C, Horiba) with an electrode (9383–10D, Horiba). The concentrations of inorganic elements and anions in the water were measured using ICP-OES (VISTA-MPX, Varian) and ion chromatography (ICS-1500, Dionex) equipped with anion exchange column (IonPac AS4A-SC, Dionex), respectively. The collected spring water was filtered using a 0.20 μm pore membrane filter (28HP-020AN, Advantec) into a plastic tube, immediately acidified with 1/50 volume 3 M HCl, and stored. The concentrations of dissolved Fe(II) and ammonium (NH_4_^+^) ions were determined by colorimetry using a phenanthroline method (Saywell and Cunningham 1937) and an indophenol blue method (Scheiner 1976), respectively. Ammonium concentrations were also checked using an ammonium electrode (5002S-10C, Horiba) with a potentiometer (D-55, Horiba). Alkalinity, the capacity of water to resist acidification, was determined using an acid-base titration with 0.05 M H_2_SO_4_ and a micro-pipette. Based on these obtained geochemical and temperature data, the dissolved inorganic carbon (DIC) concentration and *p*CO_2_ in the spring water were calculated using the computer program PHREEQC ver. 3 (Parkhurst and Appelo 2013) with a thermodynamic database “phreeqc.dat.”

**Table 1.** Water chemistry in the Sambe hot spring water and the chemical compositions in a conventional MWMM and a newly developed Sambe customized medium (SCM) (above); chemical compositions in freeze-dried Fe precipitates collected at the Sambe hot spring site (bottom).

*Liquid phase*	Sampling spot / Medium type	Na	Mg	K	Ca	Fe(II )	NH_4_	PO_4_	Cl	SO_4_	DO	DIC	pH	Temperature	Alkalinity	EC
		(mM)	(mM)	(mM)	(mM)	(μM)	(μM)	(μM)	(mM)	(μM)	(μM)	(mM)		(°C)	(meq/L)	(mS/cm)
**Spring water**	*February 26, 2022*															
	**Spot 1**	14.7	1.81	1.30	2.70	101	3.2	23.5	36.8	160	58	19.9	5.9	33	6.0	3.39
	**Spot 2**	15.1	1.84	1.33	2.80	134	3.2	22.1	36.5	180	149	12.9	6.2	29	6.0	3.39
	*December 12, 2022*															
	**Spot 1**	14.1	1.81	1.32	2.79	167	3.5	31.5	35.6	179	60	14.2	6.0	32	5.9	3.37
	**Spot 2**	14.5	1.77	1.29	2.71	71	3.1	29.3	36.1	175	126	11.8	6.2	28	5.6	3.34
**Medium**	**SCM** (This study)	15	1.8	1.4	2.8	*	187	290	13	20	*	12	5.9–6.2	28	5.7–6.0	3.2
	**MWMM** (Emerson and Moyer 1997)	5.0	0.81	0.57	0.68	*	18 700	290	20	810	*	5.0	6.2–6.4	21	11.6–12.1	3.9
***Solid phase***	**Sampling spot**	**Na**	**Mg**	**K**	**Ca**	**Fe**	**Si**	**P**	**C**	**N**						
		**(wt%)**	**(wt%)**	**(wt%)**	**(wt%)**	**(wt%)**	**(wt%)**	**(wt%)**	**(wt%)**	**(wt%)**						
**Fe precipitates**	*December 12, 2022*															
	**Spot 1**	0.27	0.09	0.11	1.5	58	0.23	2.9	0.60	0.06						
	**Spot 2**	0.40	0.10	0.11	1.4	45	0.09	1.7	0.77	0.07						

* The concentrations of dissolved Fe and O_2_ are not stated because they vary with the medium depth in a tube as shown in Fig. 4.

A part of collected Fe precipitates was immediately frozen at the laboratory upon arrival and stored at −80°C for phylogenetic analysis. An X-ray diffraction (XRD) analysis of the Sambe Fe precipitates was performed to identify their mineralogical characteristics. The frozen samples were directly freeze-dried at −50°C under vacuum condition at 20 Pa for 48 h using a freeze-dryer (DC400, Yamato Scientific). After freeze-drying, the powder sample was mounted on a glass holder and analyzed using an XRD spectrometer (Ultima IV, Rigaku) with a step size of 0.02° and a time interval of 1 s. In addition, Fe K-edge X-ray absorption near-edge structure (XANES) spectra of the Sambe Fe precipitates were also measured at beamline BL12C at KEK-PF (Tsukuba, Japan) to characterize the Fe species in the precipitates. The energy was calibrated by defining the pre-edge peak maximum of hematite as 7.113 keV. All spectra, including model compounds, were collected in transmission mode with ion chambers. Measurements were carried out at room temperature (ca. 25°C) under ambient air condition. In addition, the constituent elements in the Fe precipitates were measured using ICP-OES after digesting the dried Fe precipitates with concentrated HCl and HNO_3_. The total C and N contents in the dried Fe precipitates were quantified using a CHN elemental analyzer (JM10, J-Science Lab).

### Cultivation medium

**Figure 2.**
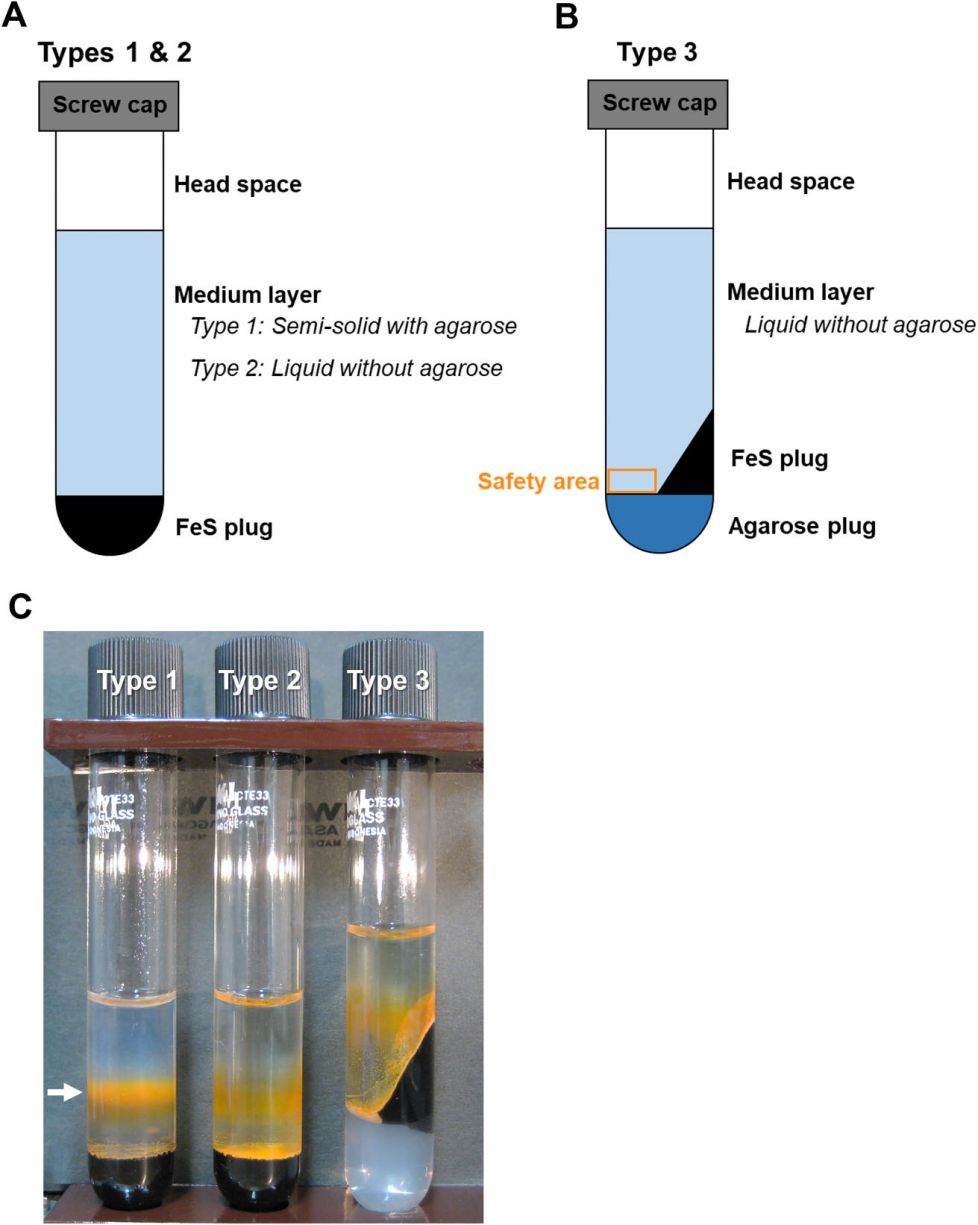
(A) Schematic figure showing medium Types 1 and 2. (B) Schematic figure of medium Type 3. (C) Photograph of the Types 1, 2, and 3 after an abiotic 7 days incubation. A white arrow in (C) indicates a distinct Fe(III) band typically observed in the conventional medium Type 1.

In this study, we prepared a Sambe-custom medium (SCM) simulating the primary elemental composition of the Sambe hot spring water (Table 1). The SCM contains 0.37 g MgCl_2_∙6H_2_O, 0.18 g NaCl, 0.060 g KCl, 0.42 g CaCl_2_∙2H_2_O, 0.050 g K_2_HPO_4_, 0.01 g NH_4_Cl, and 1.0 ml Wolfe’s trace mineral solution (ATCC MD-TMS) per liter Milli-Q^®^ water. Conventionally, an agarose-based semi-solid tube medium with a solid Fe(II) source (FeS or FeCO_3_) at the bottom (Type 1 in Fig. 2A) has been used as described in the Introduction section. The O_2_ is provided from headspace air. The semi-solid nature results in an opposing diffusion gradient of Fe(II) and O_2_ with the depth of the medium. Our strategy is to create an optimal environment near the bottom Fe(II) source to simulate the on-site O_2_ (58–149 μM) and Fe(II) (71–167 μM) levels. We first tested a liquid medium without agarose (Type 2 in Fig. 2A) to disturb the stable diffusion gradients of dissolved O_2_ and Fe(II) in the Type 1. However, the agarose-free Type 2 would not allow the unicellular Fe(II)-oxidizers to stay at a depth, and most of the enriched microbes would deposit to the bottom and contact the anaerobic Fe(II) source, which is unsuitable for aerobic Fe(II)-oxidizers. To address this concern, we prepared another type medium (Type 3) with a space (= safety area) beside the bottom Fe(II) source (box in Fig. 2B).

All types of media were prepared in 10 ml glass test tube (tube height: 100 mm, outer diameter: 15 mm, inner diameter: 9 mm; TST-SCR16-100, Iwaki) with a screw cap (9998CAP415-15, Iwaki) using the SCM. The FeS was used as the solid Fe(II) source and synthesized as described by Hanert (2006). Types 1 and 2 were prepared as described by Emerson and Floyd (2005), with minor modifications. The FeS precipitate suspension was mixed with the same volume of SCM and 1.3 wt% agarose (A9539-250 G, Sigma) in a flask. In a separate flask, a medium-layer solution was prepared in the SCM with 0.15 wt% agarose for Type 1 and without agarose for Type 2. The FeS suspension and medium-layer solutions were autoclaved at 121°C for 20 min. Subsequently, a 0.8 ml molten FeS was carefully added to the bottom of a presterilized tube in a bench and cooled at room temperature for 15–20 min to form a solid plug. When the autoclaved medium solution cooled to approximately 45°C, 10 ml of 10 wt% NaHCO_3_ solution/litter for DIC tuning and 1 ml of mixed-vitamin solution (ATCC MD-VS)/litter were added to each medium-layer solution (Emerson and Floyd 2005). Subsequently, the medium-layer solution was bubbled with filter-sterilized CO_2_ gas at 0.1 MPa through a cannula, and was set to the on-site pH 5.9–6.2 and alkalinity 5.7–6.0 meq/l. Then, 5.0 ml of this solution was carefully added on the surface of the FeS plug and capped with butyl rubber septum. The medium Type 3 requires an extra step before the preparation of FeS plug. A 1-2 ml of autoclaved (molten) SCM with 0.5 wt% agarose was added in the bottom of the tube and cooled until it solidified for ca. 30 min. After that, a 0.5–0.8 ml molten FeS was added to the bottom of tube tilted ca. 45° and cooled until it solidified. Thereafter, 4.0 ml of the prepared medium solution (without agarose) was added, as with the Type 2 medium.

To validate the prepared Types 1–3 media, depth profiles of dissolved Fe(II) and DO were obtained because these vary with depth in medium-layer. The depth profiles of DO in the medium-layer were determined using an O_2_ microelectrode (tip diameter of 40–60 μm; OX-50, Unisense). A two-point calibration was made in aerated and deoxygenated water at incubation temperature (28°C). The profiles were obtained by vertically placing the microelectrode into the medium using a PC-controlled micromanipulator. An arm-mounted microscope was used to visually determine the contact point between the microelectrode tip and surface of the medium layer (depth zero). The dissolved Fe(II) was examined at three depths in the medium layer: surface, middle, and bottom layers. Using a syringe with a long needle, medium solution (0.5 ml) was carefully collected from each layer, immediately filtered through a membrane filter, acidified by the addition of 1/50 volume of 3 M HCl, and stored in a refrigerator prior to analysis. Total Fe and Fe(II) concentrations in the solution was determined by the ICP-OES and phenanthroline method, respectively. Our preliminary experiments showed that the total Fe and Fe(II) concentrations in the medium were almost equal, and no dissolved Fe(III) was detected.

### Enrichment and isolation

Inoculation was performed by dispensing serial 10-fold diluted solutions of the collected Fe precipitates with a sterile pipette. The collected precipitates were diluted with autoclaved agarose-free SCM solution, and the dilution factor in the inoculation ranged from 10^−4^ to 10^−6^. The inoculation volume was 100 μl per tube. All media types were incubated in the dark incubator at a constant temperature (28 ± 1°C) for 14 days. In the enrichment tubes in which the target *Gallionellaceae* members were successfully dominated, the Fe precipitates in the tube were collected and diluted to 10^−7^ in 10-fold serial dilutions for the isolation. Each dilution was inoculated into a fresh medium. After four transfers, the cultures were tested for the presence of heterotrophic microorganisms by streaking the cell suspensions onto R2A and 10% R2A agar plates.

### DNA extraction from Fe flocs and its phylogenetic analysis

Microbial DNA was extracted from the Fe precipitates collected at the spring site using a commercial kit (Extrap Soil DNA Kit Plus ver.2, BioDynamics Laboratory Inc.). The DNA was finally eluted with 50 μl Tris–EDTA (TE) solution and stored at 5°C prior to further analysis. The bacterial community structure in the Fe precipitates was estimated using high-throughput amplicon sequencing of the microbial 16S ribosomal RNA (rRNA) gene. The V3–V4 region of the 16S rRNA gene was amplified from the DNA samples using the primer set U341F and U805R (Herlemann et al. 2011) and KOD FX Neo DNA polymerase (KFX-201, Toyobo) in buffer. The primer sets used are summarized in Supplementary Table S1. For amplification, an Illumina adaptor sequence (ACACTCTTTCCCTACACGACGCTCTTCCGATCT) and Illumina Multiplexing PCR primer 2.0 sequence (GTGACTGGAGTTCAGACGTGTGCTCTTCCGATCT) were added at the ends of the primers. The PCR amplification conditions consisted of denaturation at 94°C for 2 min and 25 to 28 amplification cycles of denaturation at 98°C for 10 s, annealing at 55°C for 30 s, and elongation at 68°C for 30 s. The amplicons were mixed with Illumina PhiX control libraries and sequenced using the Illumina MiSeq platform (Illumina) with 2  ×  300-bp paired-end reads. Amplicon sequence data were processed using the following procedure. Raw paired-end reads were merged using FLASH ver. 1.2.11 (Magoč and Salzberg 2011), and primer sequences were removed using FASTX-Toolkit ver. 0.0.14 (Hannon 2010). Low-quality (Qscore < 20) and short (< 130 bp) reads were filtered out using Sickle ver. 1.33 (Joshi and Fass 2011). The resulting sequences were analyzed using QIIME2 pipeline ver. 2023.2 (Bolyen et al. 2019). Unique amplicon sequence variants were generated using the DADA2 plugin wrapped in QIIME2, and chimeric sequences were removed (Callahan et al. 2016). The sequences were clustered into amplicon sequence variants (ASVs) with 97% sequence identity in each library. The taxa were assigned to ASVs for the 16S rRNA genes using the QIIME2 plugin feature-classifier classify-sklearn (Bokulich et al. 2018) against the SILVA ver. 138 database (Quast et al. 2013).

The abundance of the *Gallionellaceae* members in the enrichments was estimated using quantitative PCR (qPCR) technique to evaluate the enrichment efficiency among media types. After 14 days incubation, the Fe precipitates in the medium were collected from the Fe growth band (Type 1), the bottom of medium-layer (Type 2), and the safety area (Type 3) with a sterile pipette tip, and microbial DNA was extracted from collected precipitates as described above. The qPCR was performed with a master mix (Kapa SYBR fast qPCR, Kapa Biosystems) using a real-time PCR system (StepOnePlus, Applied Biosystems). The PCR amplification was performed in a 20 μl reaction system with ∼0.5 ng of DNA and 0.5 μM specific primers for the *Gallionellaceae* family (Ashelfold et al. 2002, Naruse et al. 2019) and bacterial universal 16S rRNA genes (Suzuki et al. 2000). The details are summarized in Supplementary Table S1. The PCR amplifications were conducted using the following program: (i) 95°C for 5 min, (ii) primer-specific cycles of 95°C for 45 s, primer-specific annealing temperature for 45 s, then 72°C for 30 s, (iii) final elongation at 72°C for 7 min, and (iv) melt curve stage from 60°C to 95°C. The enrichment efficiency was evaluated using *Gallionellaceae* / bacterial 16S rRNA genes ratio. For the qPCR analysis, standard curves were drawn from a tenfold serial dilution between 1 × 10^1^ and 1 × 10^8^ copies per reaction. Standard DNA for qPCR were generated using the M13 primer set from a plasmid containing the target sequences of the *Gallionellaceae* specific and universal bacteria 16S rRNA genes.

### Growth of isolated strains

Cells of isolated strains were stained with 0.1 mM Syto9 (Invitrogen) as nucleic acid-binding dye and visualized using an epifluorescence microscope (BX-51, Olympus) with a digital camera (EOS Kiss X4, Canon). The cell growth rate was determined by direct cell counting method (Emerson and Moyer 1997). The isolated strain was inoculated at the same time with identical amounts of cell suspension and incubated together. Every three days, the tubes were vortexed for 30 s to homogenize the medium-layer solution. For cell counts, a 10 μl aliquot of the homogenized solution was smeared in defined circles on an aminopropyltriethoxysilane-coated glass slide and allowed to air-dry for an hour. The Syto9-stained cells in all 15 microscope fields were counted at × 1000 magnification using the epifluorescence microscope. For the quantification of oxidized Fe (Fe(III)) species during the growth, a 1.0 ml aliquot was collected from the homogenized medium-layer and digested by adding the same amount of 3 M HCl. Total Fe and Fe(II) concentrations in the digested solution was determined using the ICP-OES and phenanthroline method, respectively. The Fe(III) fraction was estimated by subtracting Fe(II) from the total Fe, while assuming Fe(II) and Fe(III) as the possible Fe species.

The optimum pH and temperature for the isolates were determined according to previously described methods (Krepski et al. 2012, Chiu et al. 2017). The pH range for growth was tested by buffering sets of media to different pH levels. A 10 mM acetate buffer was used at pH 5.0. A 10 mM MES adjusted with NaOH was used to buffer the pH 5.5, 6.0, and 6.5. A 5 mM HEPES adjusted with NaOH was used to buffer the pH 7.0 and 7.5. The pH measurements taken before and after cultivation confirmed minimal decreases (0.1–0.2) during cultivation periods. Optimum growth temperature was determined by incubating cultures at 5, 10, 15, 20, 25, 30, 35, and 40°C. All cultivations with Type 3 medium were assessed for growth after one and two weeks based on direct cell counting with fluorescence microscopy, as described above.

### Draft genome analyses of isolates

For DNA extraction, strains UT4 and UT5 were grown in the Type 3 medium (50 ml volume per strain) with FeS plug. Genomic DNA was extracted from these cultures using the above method. The DNA quality and concentration were confirmed using a NanoDrop Lite (Thermo Scientific) before sequencing. Genome sequencing was performed using a DNBSEQ-G400 sequencer with 2  ×  150-bp paired-end reads. The reads were filtered using fastp version 0.23.4 (Chen 2023). High-quality reads were then assembled using Shovill version 1.1.0 (https://github.com/tseemann/shovill). Annotation of the assembled contigs with 500 bp or longer were performed using DFAST version 1.2.20 (Tanizawa et al. 2018), Kyoto Encyclopedia of Genes and Genomes (KEGG) (Kanehisa et al. 2017) with BlastKOALA (Kanehisa et al. 2016), and FeGenie version 1.2 (Garber et al. 2020). Annotation of the assembled contigs with 500 bp or longer were performed using DFAST version 1.2.20 (Tanizawa et al. 2018), Kyoto Encyclopedia of Genes and Genomes (KEGG) (Kanehisa et al. 2017) with BlastKOALA (Kanehisa et al. 2016), and FeGenie version 1.2 (Garber et al. 2020). Values of average nucleotide identity (ANI) and average amino acid identity (AAI) among the genomes were calculated using online tools (Rodriguez-R and Konstantinidis 2016). A phylogenomic tree of concatenated sequences of the bacterial 120 marker proteins defined by Genome Taxonomy Database (GTDB) (Parks et al. 2022) was constructed as described previously (Kato et al. 2022) with minor modifications. The alignment of the concatenated protein sequences was generated using GTDB-tk version 2.4.0 (Chaumeil et al. 2022) with the reference database R220, and used for the Maximum Likelihood tree reconstruction by IQ-TREE version 2.3.6 with the substitution model LG+F+I+R5. Taxonomic classification of the obtained genomes was also performed using GTDB-tk based on relative evolutionary divergence (RED).

## Results and discussion

### Chemical and microbial analyses of natural sample

The physicochemical properties of the spring water are summarized in Table 1 (above). The water temperature was mesothermic 28−33°C and the pH was 5.9−6.2 at spots 1 and 2. The weakly acidic pH is a result of the Sambe hot spring being a carbonated spring (Shiraishi et al. 2019), which was also supported by the high alkalinity and DIC obtained in our study (Table 1). On-site dissolved O_2_ values (58−149 μM) were roughly a half to quarter of the atmospheric equilibrium concentration (220−240 μM) at the site temperature and salinity (ca. 3‰). Dissolved Fe(II) in the water ranged from 71–167 μM with no detectable dissolved Fe(III) (< 0.2 μM). The concentrations of primary cations, Na^+^, K^+^, Mg^2+^, and Ca^2+^ through spots were 14.1–15.1 mM, 1.29–1.33 mM, 1.77–1.84 mM, and 2.70–2.80 mM, respectively. The concentrations of primary anions, SO_4_^2−^, Cl^−^, and PO_4_^3−^ were 0.16–0.18 mM, 36.1–36.8 mM, and 22.1–31.5 μM, respectively. Low concentrations of NH_4_^+^ ions (3.1–3.5 μM) were detected, whereas the oxidized nitrogen species (nitrite and nitrate ions) were not detected. Relatively high concentrations of Na^+^, Mg^2+^, and Cl^−^ ions, consistent with a high EC (ca. 3.3 mS/cm), are a typical feature of hydrothermal spring water (White 1957, Langmuir 1997, Stumm and Morgan 2012). The collected Fe precipitates contained Fe as the most abundant element (45–58 wt% dry, Table 1). The XRD patterns of the spots 1 and 2 precipitates show two broad reflections with no sharp peaks (Supplementary Fig. S1). These findings indicate that the precipitates mainly consist of poorly crystalline Fe(III) oxyhydroxides (e.g. 2-line ferrihydrite), which was confirmed by the Fe XANES spectral features of the Sambe Fe precipitates (Supplementary Fig. S2) and a previous report on this site (Mitsunobu et al. 2012). The total number of microbial cells in the Fe precipitates (collected at spot 2) ranged from 2.8 × 10^8^ to 5.1 × 10^8^ cell/ml.

In this study, we prepared a custom-made medium (SCM) that simulated the composition of primary elements, especially Na, Mg, K, Ca, DIC, and pH in Sambe spring water (Table 1). This was done because (i) site-specific ionic concentrations would be ideal for cultivation and (ii) the chemical composition of this spring water was significantly different from that of conventional MWMM as clearly shown in Table 1. In the preparation, the elemental composition of spot 2 was targeted. The DIC (12 mM), alkalinity (5.7–6.0 mM), and pH (5.9–6.2) in the SCM were adjusted by NaHCO_3_ addition and CO_2_ bubbling following the thermodynamic calculation of spring water (Table 1). Although relatively small N and P, which are biologically essential elements, were observed in the spring water, certain amounts were contained in the solid phase Fe precipitates (P: 1.7–2.9 wt%, N: 0.06–0.07 wt%) (Table 1 bottom). Thus, larger P (10 times) and N (60 times) than in the spring water were added in the phosphate and ammonium forms, respectively. Sulfur in the medium was below the on-site level, expecting the passive addition from the solid Fe(II) source (FeS) during incubation. The measured EC of the SCM (3.2 mS/cm) was similar to the on-site EC levels (3.3–3.4 mS/cm) (Table 1), indicating that the primary ionic concentration in the prepared SCM matched approximately.

**Figure 3.**
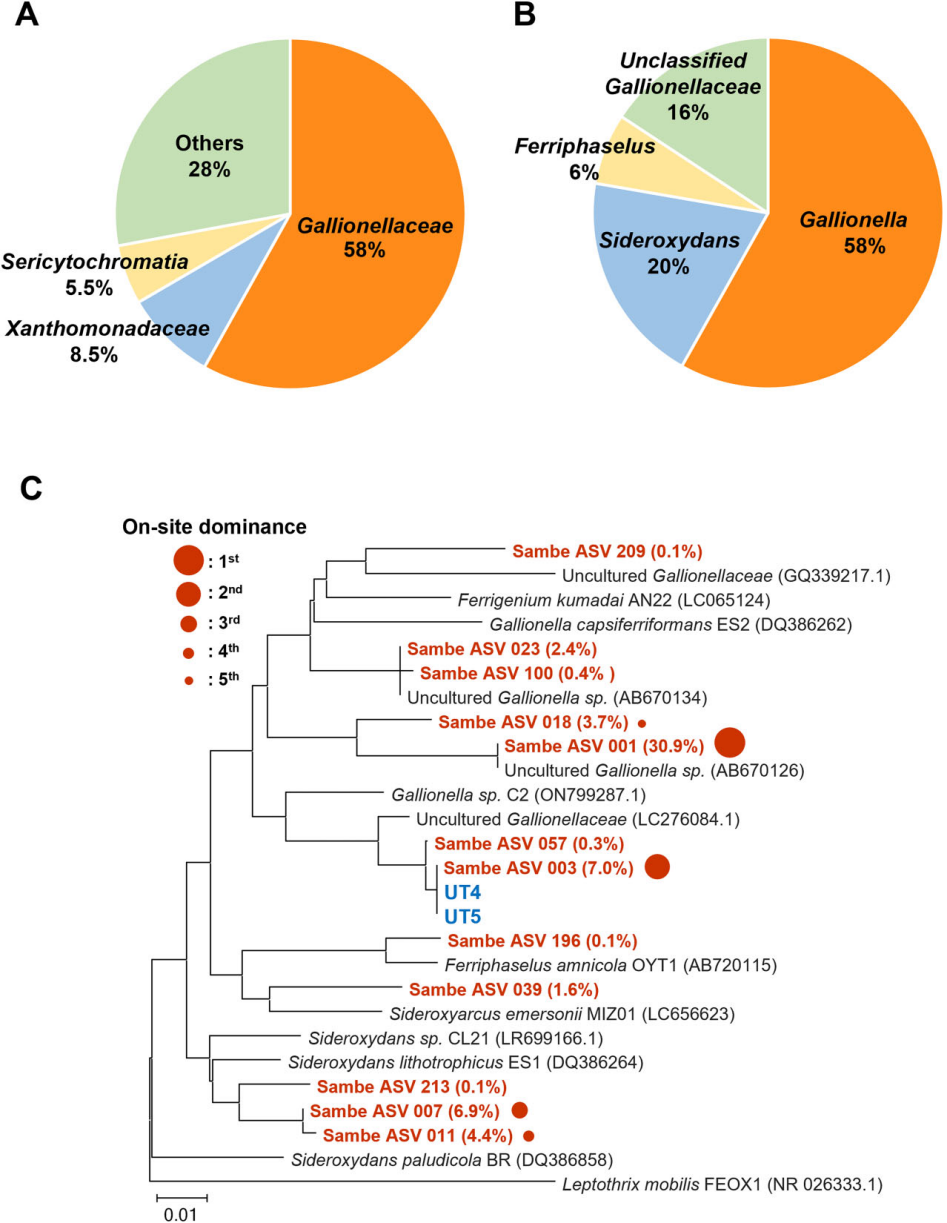
(A) Pie chart showing the bacterial community at family level in the Fe precipitate collected at the Sambe spot 2 based on 16S rRNA (V3–V4 region) amplicon sequencing. (B) Pie chart at genus level in *Gallionellaceae* of (A). (C) Phylogenetic tree of *Gallionellaceae* ASVs (dominance in the whole bacterial community > 0.1%) found in the Sambe spot 2 and strains UT4 and UT5 based on Neighbor-Joining method. *Leptothrix mobilis* was chosen as the out group. In (C), the percentage value for each ASV represents the percentage in the bacterial community (not in the *Gallionellaceae* community).

Figure 3 shows the bacterial community in the Sambe Fe oxide precipitates based on 16S rRNA gene amplicon sequencing. At the family level, *Gallionellaceae* (58%), *Xanthomonadaceae* (8.5%), and *Sericytochromatia* (5.5%) dominated the community. The *Gallionellaceae* family consisted of the genera *Gallionella* (58%), *Sideroxydans* (20%), *Ferriphaselus* (6%), and considerable amount of unclassified *Gallionellaceae* (16%). Almost all terrestrial chemolithotrophic neutrophilic Fe(II)-oxidizing bacteria isolated to date belong to the *Gallionellaceae* family (Emerson and Moyer 1997, Lüdecke et al. 2010, Krepski et al. 2012, Kato et al. 2014, Khalifa et al. 2018, Kato et al. 2022, Hoover et al. 2023). The dominance of *Gallionellaceae* is consistent with the available Fe(II) and O_2_ in the water and Fe(III) detected in the Fe precipitates in the Sambe hot spring, suggesting that *Gallionellaceae* mainly contributed to microbial Fe(II)-oxidation at this site. Therefore, this family was selected as the enrichment target in the present study.

### Oxygen and Fe profiles in enrichment media

**Figure 4.**
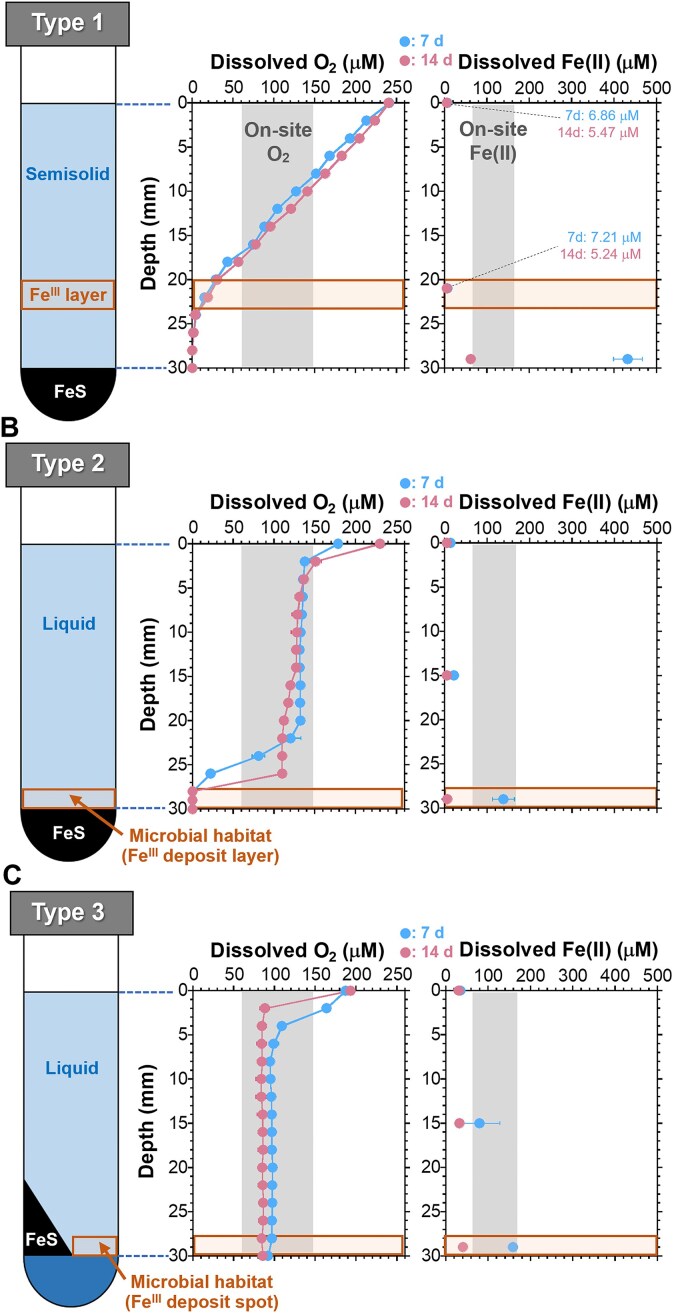
Depth profiles of dissolved O_2_ and Fe(II) in the abiotic incubation with Type 1 (A), Type 2 (B) and Type 3 (C). Gray areas in figures indicate the on-site O_2_ and Fe(II) levels. Brown box in the figure indicates the Fe(II)-oxidizer's habitat (Fe(III) deposit) layer expected in each medium type. In the figures, error bars represent 1 standard deviation from the mean.

The depth profiles of dissolved O_2_ and Fe in un-inoculated medium Types 1–3 are shown in Fig. 4. Conventional medium (Type 1) has a distinct Fe(III) oxide layer in the middle (white arrow in Fig. 2C; Emerson and Floyd 2005, Kato et al. 2013, Chiu et al. 2017). Growth colonies of Fe(II)-oxidizers are typically observed near the Fe(III) layer in the medium (Emerson and Floyd 2005, Kato et al. 2013, Chiu et al. 2017) because of comparable biotic and abiotic Fe(II) oxidation rates (Singer and Stumm 1970, Roden et al. 2004). The dissolved O_2_ concentration, which was atmospheric equilibrium concentration (∼240 μM) at the surface, gradually decreased with the depth drawing a diffusion gradient (Fig. 4A) and ranged between 10–30 μM in the Fe(III) layer. The Fe(II) concentrations in the Fe(III) oxide layer were low (5–7 μM) through the incubation period, whereas higher in the bottom layer (Fig. 4A). This suggests that the medium Type 1 did not provide a favorable habitat that satisfied both the on-site O_2_ and Fe(II) levels around the Fe(III) oxide layer (Fig. 4A).

In the Type 2 agarose-free liquid medium, most of enriched microbes are expected to deposit on the FeS surface with particulate Fe(III) oxides. Compared with that of Type 1, the O_2_ diffusion gradient was almost broken in the medium layer and locally formed near the surface of the FeS plug (Fig. 4B). Since the FeS is a strong reductant, the O_2_ drastically decreased to nearly zero at the FeS surface (expected microbial habitat) with on-site Fe(II) (101–145 μM). Hence, the medium Type 2 provides defective O_2_ and matched Fe(II) in the (expected) microbial habitat.

To address this matter, we prepared a new medium Type 3 having a safety area for enriched microbes by modifying the layer structure. The medium has a small area adjacent to the FeS plug (box in Fig. 2B) as ideal oxic-anoxic interface and safety habitat. As a result, this modification worked well. The O_2_ and Fe concentrations in the safety area ranged 70–80 μM and 40–150 μM (Fig. 4C), respectively, throughout the 14 days incubation, which were close to the field O_2_ (58–149 μM) and Fe(II) (71–167 μM) levels (Fig. 4C; Table 1). These results suggest that the Type 3 medium has a higher potential to enrich the environmentally dominant *Gallionellaceae*.

### Comparison of enrichment efficiency

**Figure 5.**
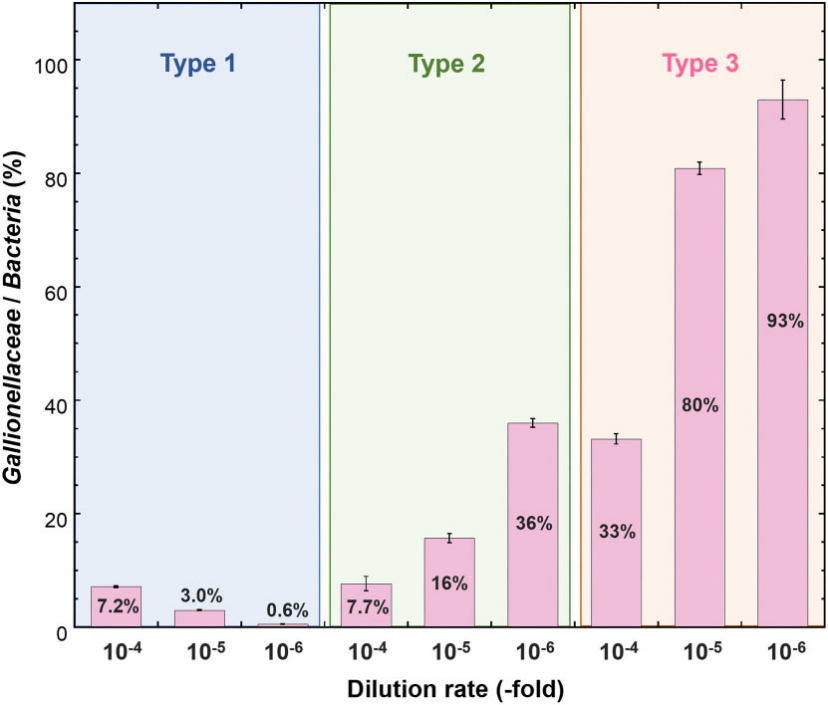
*Gallionellaceae/Bacteria* ratios after the first enrichment (14 days incubation) with medium Types 1–3 estimated by the qPCR with *Gallionellaceae* and *Bacteria* specific primers. The 10^−4^ to 10^−6^ diluted Fe precipitates were inoculated to the media.

The 10^−4^ to 10^−6^ diluted Fe precipitates were inoculated in each medium type and enriched for 14 days in the dark. After the first enrichment, we estimated the abundance of the target *Gallionellaceae* in Types 1–3 using qPCR (Fig. 5). Microbial DNA was extracted from the Fe(III) precipitates in each medium collected from the Fe growth band (Type 1) and the Fe deposit layer/spot (Types 2 and 3) in illustrations in the left side of Fig. 4. Notably, the abundance ratios are only the estimation and relative ratios of the proportion of *Gallionellaceae* in the whole bacterial population in the enrichments, while the copy number of 16S rRNA genes cannot be exactly correlated with cell numbers due to variations in the number of rRNA operons per bacterial genome (Klappenbach et al. 2000) and in the genome copy number per cell, depending on the bacterial growth rate (Maldonado et al. 1994, Pecoraro et al. 2011). In the conventional Type 1, the relative abundances of *Gallionellaceae* were 7.2% (10^−4^ dilution), 3.0% (10^−5^ dilution), and 0.6% (10^−6^ dilution) in the bacterial community (Fig. 5). In contrast, the fractions in the Type 3 were 33% (10^−4^ dilution), 80% (10^−5^ dilution), and 93% (10^−6^ dilution) (Fig. 5), which were up to 150 times higher than that of the Type 1 at the 10^−6^ dilution. The efficiency in Type 3 were also higher than that in Type 2 ranging from 7.7 to 36% (Fig. 5). In addition, the enrichment efficiency in the Type 3 with SCM were significantly higher than those of Type 3 with MWMM at all dilution rates (Supplementary Fig. S5). Thus, these results clearly indicated that the medium Type 3, in which the abundances of O_2_, Fe, and primary elements were customized to the field levels, enriched the *Gallionellaceae* with much higher proportion by just one incubation. The higher enrichment ability of the Type 3 has also been verified at other environments, Fe-containing saline groundwater and hot spring sites (Table S3 and Fig. S4 in Supplementary material), suggesting the higher ability of the custom-made approach across the systems. Noticeably, the *Gallionellaceae* proportion in the Type 3 increased with the higher dilution factors (Fig. 5) though the proportion in the Type 1 decreased with the dilution. This is possibly owing to the lower numbers of competitors and organic substances (nutrients for heterotrophs) in the higher-dilution tubes. These results suggest that the Type 3 provided a preferable condition for growth of the *Gallionellaceae* microbes. In particular, the modification of removing agarose, a potential substrate for heterotrophs, might be more “effective” in enriching the chemolithotrophic Fe(II)-oxidizing bacteria by limiting microbial contaminants. The isolation was performed using the 10^−6^ dilution of Type 3, which showed the highest *Gallionellaceae* abundance.

### Physiological and phylogenetic characterization of isolated strains

**Figure 6.**
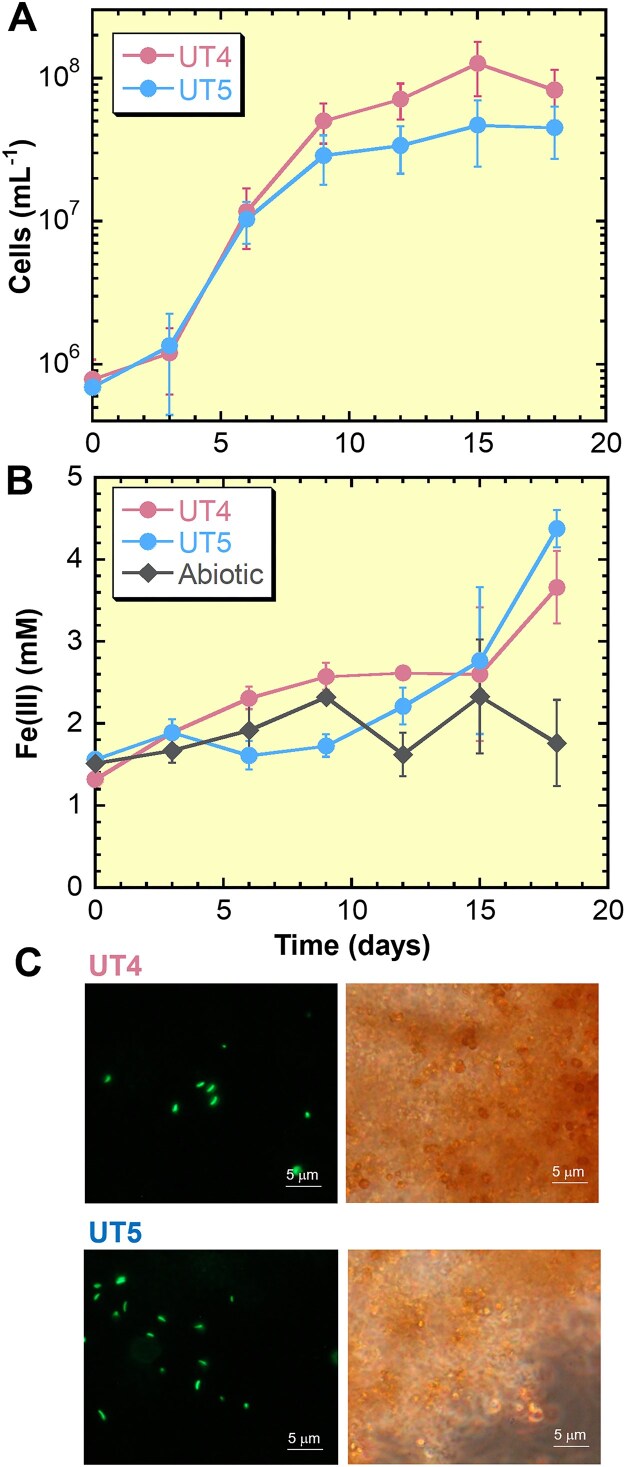
Growth curves (A) and total Fe(III) curves (B) for strains UT4 and UT5 cultivated in medium Type 3 with FeS plug at 28°C. Abiotic control (no-inoculation) was shown in (B). In (A) and (B), error bars represent 1 standard deviation from the mean. (C) Fluorescence images of UT4 and UT5 cells (left side) and phase contrast images (right side) of the same field of fluorescence images.

*Gallionellaceae* strains UT4 and UT5 were successfully isolated from independent enrichments after three transfers of the 10^−7^ serial dilution. Purity was demonstrated by the lack of growth on R2A medium (no contaminant oligotrophs). Microscopic observations showed that both UT4 and UT5 were curved-rod shape and 1.4–1.8 µm in length (Fig. 6C), which is consistent with studies on the Gallionellaceae family*, Gallionella, Sideroxydans, Ferriphaselus, Ferrigenium, Sideroxyarcus* (Emerson and Moyer 1997, Lüdecke et al. 2010, Krepski et al. 2012, Khalifa et al. 2018, Kato et al. 2022). Both strains did not form the stalk-like materials and was instead associated with the particulate Fe hydroxides (Fig. 6C). The UT4 and UT5 cell counts (Fig. 6A) in medium Type 3 showed distinct growth curves with Fe(III) increase and doubling times of 22 h (UT4) and 25 h (UT5), respectively. In addition, both strains grew in the conventional medium (Type 1) but the growth rates were 5–6 times slower, with doubling times of 119 h (UT4) and 147 h (UT5) (Supplementary Fig. S3), which also supports the fact that medium Type 3 has a superior efficiency in enriching the Sambe *Gallionellaceae*. Previous physiological studies of *Gallionellaceae* isolates have shown that these Fe(II)-oxidizing bacteria prefer microaerophilic environments. The *Gallionellaceae* members are often found in environments with dissolved O_2_ concentrations of <5% of air-saturated values (<25 μM) and Fe(II) concentrations of tens to several hundreds μM (Emerson and Revsbech 1994, Emerson and Moyer 1997, Kato et al. 2013), and it is recommended that these conditions be simulated in the cultivation medium (Emerson et al. 2005). The conventional Type 1 medium are superior at reproducing these conditions (Emerson et al. 1994, Kato et al. 2013), leading to the isolation of several strains (Emerson and Moyer 1997, Lüdecke et al. 2010, Kato et al. 2014, Khalifa et al. 2018, Kato et al. 2022, Hoover et al. 2023). Laboratory experiments with isolates (strains ES-1 and BrT of the genus *Sideroxydans*) have shown that microbial oxidation is comparable to or higher than abiotic oxidation (20%–90% of total Fe oxidation) with O_2_ concentration of 10–50 μM (Neubauer et al. 2002, Druschel et al. 2008). They suggest that in such microaerophilic environments, the rate of abiotic Fe(II) oxidation is relatively low and thus the Fe(II)-oxidizing bacteria can compete with the abiotic oxidation. In this study, new *Gallionellaceae* species (strains UT4 and UT5) were isolated by matching the dissolved O_2_ and Fe(II) in the culture medium (O_2_: 70–80 μM, Fe(II): 40–150 μM; Fig. 4C) to the on-site levels (O_2_: 58–149 μM, Fe(II): 71–167 μM; Table 1). While this O_2_ level is line with the field condition, it is 1.6 to 8 times higher than the range mentioned above (10–50 μM). Both UT4 and UT5 strains grow very slowly in the conventional Type 1 medium (Fig. S3), which suggests that the O_2_ and Fe(II) levels reproduced in our customized Type 3 medium are more suitable for the growth. Thus, our findings indicate a possibility that there are Fe(II)-oxidizing bacteria for which low O_2_ (<50 μM) that compete with the abiotic oxidation are not optimal condition for the growth. In natural environments abundant in Fe(II) and O_2_ (where energy substrates are not severely limited), it may be more important than overcoming abiotic Fe(II) oxidation to adapt to the on-site condition and compete for survival with other microorganisms. In fact, the *Gallionellaceae* Fe(II) oxidizers are the most dominant prokaryotic microorganism in this Sambe hot spring site (Fig. 3A).

**Figure 7.**
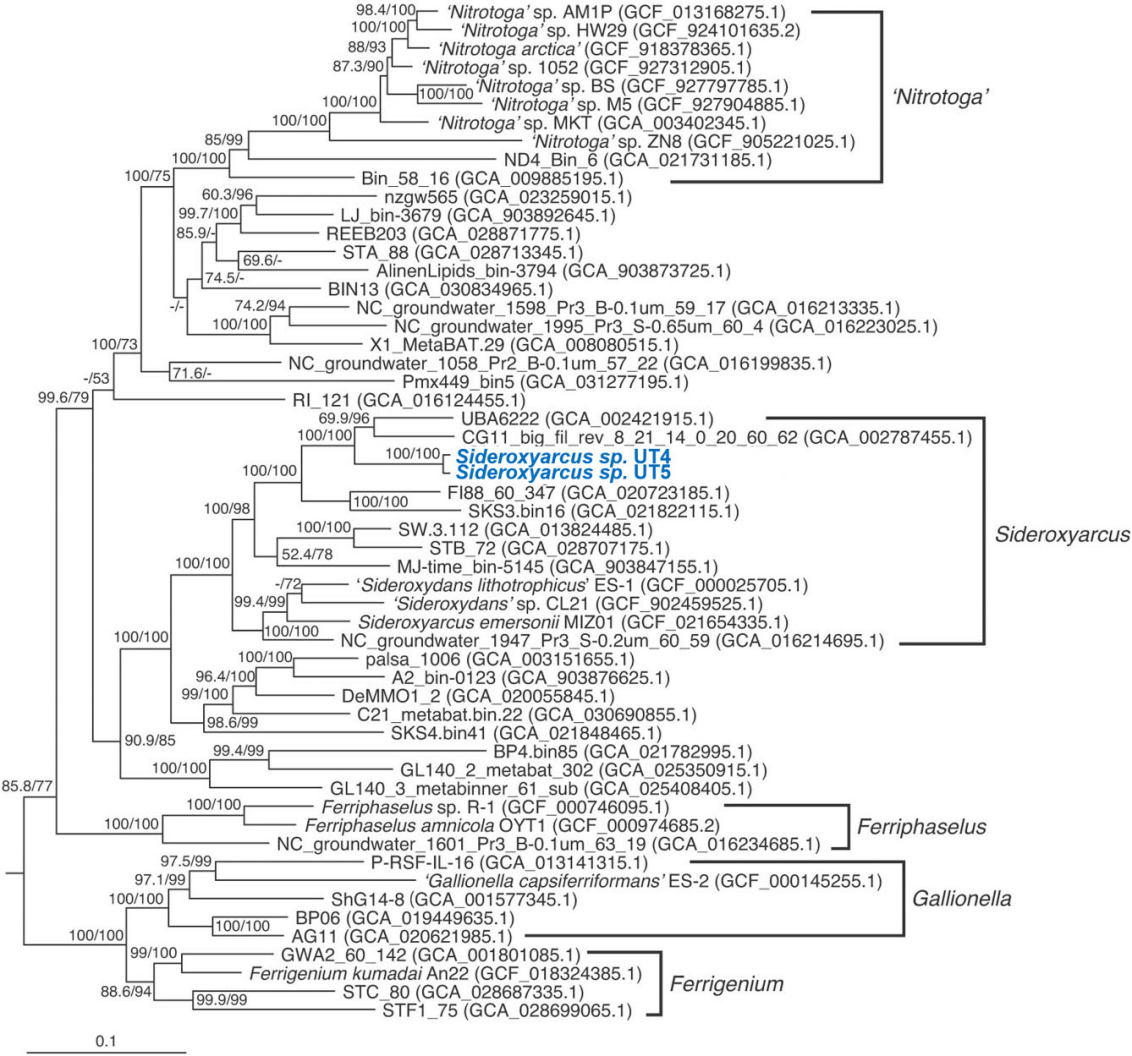
Phylogenomic tree of the family *Gallionellaceae*. The genus-level clades were indicated based on the GTDB taxonomic classification, except for the genus *Ferrigenium*. The strains UT4 and UT5 were indicated in bold. Values of 1000 replicates for SH-aLRT (left, %) and ultrafast bootstrap (right, %) were indicated at the nodes (<50% are not shown). The sequences of *Nitrosomonas mobilis* Ms1 (GCF_900103035.1), *Nitrosomonas cryotolerans* ATCC 49181 (GCF_900143275.1), and *Nitrosospira multiformis* ATCC 25196 (GCF_000196355.1) were used as the outgroup (not shown).

The preferred pH and temperature ranges of both strains were pH 5.5–6.5 and 25–35°C, respectively, which consistently overlapped with the on-site ranges (pH 5.9–6.2 and 28–33°C; Table 1). Moreover, the draft genomes of both strains possessed key functional genes, *cyc2* gene encoding a putative Fe(II) oxidase and ribulose-1,5-bisphosphate carboxylase oxygenase (RuBisCO) gene encoding the Calvin-Benson-Basham cycle, a carbon fixation pathways (Supplementary Table S2). Overall, our experiments suggest that both UT4 and UT5 are chemolithotrophic Fe(II)-oxidizing bacteria, which is consistent with almost all *Gallionellaceae* isolates. Among all other *Gallionellaceae* isolates, strains UT4 and UT5 were the most similar to each other based on ANI (98.7%) and 16S rRNA gene identity (99.9%) (Table 2), indicating that the two strains belong to the same species. The UT4 and UT5 strain genomes are 2.15 and 2.22 Mbp, respectively. The sizes are slightly smaller than those of other *Gallionellaceae* isolates (2.6–3.2 Mbp) (Kato et al. 2022) and MAGs (2.6–3.4 Mbp) (Bendall et al. 2016, He et al. 2016, Kadnikov et al. 2016, Chan et al. 2023). Because both strains share less than 98.7% 16S rRNA identity (Stackebrandt and Ebers 2006) and have less than 95% ANI (Konstantinidis and Tiedje 2005) compared with those of other *Gallionellaceae* isolates (Table 2). These values supported that strains UT4 and UT5 should represent a new species of the genus *Sideroxyarcus*. Indeed, the AAI values with *Sideroxyarcus emersonii* were ca. 74% (Table 2), which was higher than the genus-level boundary of 65%–72% (Konstantinidis and Tiedje 2007). Moreover, both strains were classified as “d_Bacteria; p_Pseudomonadota; c_Gammaproteobacteria; o_Burkholderiales; f_Gallionellaceae; g_Sideroxyarcus; s_” by GTDB-tk. The phylogenomic tree (Fig. 7) indicated that both strains were clustered with the other *Sideroxyarcus* spp. It should be noted that some isolates with the invalid genus name “*Sideroxydans*” could be affiliated in the genus *Sideroxyarcus* as reported previously (Kato et al. 2022). In comparison with bacterial community in the hot spring precipitates (Fig. 3C), both isolates were the closest to Sambe ASV 003. The ASV 003 was the second most environmentally-dominant species (7.0%) in the Sambe *Gallionellaceae* community (Fig. 3C). More detailed physiology (e.g. optimum salinity, substrate utilization) and genomics of the UT4 and UT5 will be compiled in a separate paper and are not addressed in this paper that mainly deals with the newly developed custom-made medium approach. In addition, the missing factors necessary for the enrichment of the most dominant environmental *Gallionellaceae* species (e.g. Sambe ASV 001), which was not successful in this study, will be the subject of future work.

**Table 2.** 16S rRNA gene identities and ANI/AAI comparisons of strains UT4 and UT5 to other *Gallionellaceae*.

Name	16S rRNA gene identity (%)		ANI (%)		AAI (%)		Reference
	Strain UT4	Strain UT5	Strain UT4	Strain UT5	Strain UT4	Strain UT5	
*Sideroxyarcus sp*. UT4	100	99.90	100	98.69	100	98.53	This study
*Sideroxyarcus sp*. UT5	99.90	100	98.69	100	98.53	100	This study
*Sideroxydans paludicola* BrT	96.10	95.78	*	*	*	*	Weiss et al. 2007
*Sideroxydans lithotrophicus* LD-1	94.11	94.14	*	*	*	*	Weiss et al. 2007
*Sideroxyarcus emersonii* MIZ01	94.16	93.23	79.81	77.64	74.35	74.29	Kato et al. 2014
*Sideroxydans lithotrophicus* ES-1	94.55	93.67	79.63	77.21	73.83	73.69	Emerson et al. 2013
*Sideroxydans sp*. CL21	94.09	93.99	79.12	76.05	72.82	72.52	Cooper et al. 2020
*Ferrigenium kumadai* An22	94.90	94.25	78.70	76.00	69.68	69.71	Watanabe et al. 2021
*Ferriphaselus sp*. R-1	92.83	92.79	78.19	75.14	67.51	67.26	Krepski et al. 2012
*Ferriphaselus amnicola* OYT1	92.39	92.50	77.20	73.59	67.65	67.38	Kato et al. 2014
*Gallionella capsiferriformans* ES-2	92.05	92.49	75.48	71.81	66.59	66.28	Emerson et al. 2013
*Candidatus Nitrotoga fabula* KNB	94.41	94.32	75.04	69.66	63.89	63.43	Keuter et al. 2022
*Candidatus Nitrotoga arctica* 6680	94.15	94.04	74.27	70.36	64.99	64.66	Keuter et al. 2022

* Genomic data are unavailable.

## Conclusions

We newly proposed a custom-made medium approach for chemolithotrophic Fe(II)-oxidizing bacteria following the specific field condition. The dissolved Fe(II) and O_2_ (as energy substrates), primary elements (Na, Mg, K, Ca, N, P, and S), pH, alkalinity, DIC, total ionic concentration (indexed to EC), and temperature in the medium were tuned by modifying the medium structure and chemical composition based on the geochemical analysis and thermodynamic calculation of environmental condition, while not fully matched for several elements. Using this new medium approach, we successfully enriched chemolithotrophic Fe(II)-oxidizing *Gallionellaceae* by just one incubation and isolated the environmentally leading new species from a natural hot spring site. This approach provides a higher enrichment efficiency for chemolithotrophic Fe(II)-oxidizing bacteria at the enrichment despite these bacteria are typically the difficult-to-culture microbes, which shortens the labor-intensive isolation process and helps to break the limitation of cultivation-dependent study of the Fe(II)-oxidizing bacteria. Furthermore, this approach following the specific environmental condition has a strong advantage for the isolation of the environmentally-leading species, which may be useful to obtain the physiological and ecological nature of the primary players in environmental Fe cycles.

## Supplementary Material

fiaf051_Supplemental_File

## Data Availability

The amplicon sequence data of natural Fe mats were deposited under BioProject accession number PRJDB18476, BioSample accession number SAMD00800822, and DDBJ Sequence Read Archive (DRA) accession number DRX563044. The draft genome sequences were deposited in DDBJ Mass Submission System (MSS) under accession numbers BAAFRX010000001-BAAFRX010000062 (UT4), BAAFRY010000001-BAAFRY010000075 (UT5), LC833333 (16S rRNA sequence of UT4), and LC833334 (16S rRNA sequence of UT5), BioProject accession numbers PRJDB18493 (UT4) and PRJDB18495 (UT5), BioSample accession numbers SAMD00802309 (UT4) and SAMD00802315 (UT5), and DRA accession numbers DRX563096 (UT4) and DRX563097 (UT5).

## References

[bib1] Appelo CAJ, Postma D. Geochemistry, Groundwater and Pollution(2nd ed.). London: CRC Press, 2005.

[bib2] Ashelford KE, Weightman AJ, Fry JC. PRIMROSE: a computer program for generating and estimating the phylogenetic range of 16S rRNA oligonucleotide probes and primers in conjunction with the RDP-II database. Nucleic Acids Res. 2002;30:3481–9. 12140334 10.1093/nar/gkf450 PMC137075

[bib3] Bendall ML, Stevens SLR, Chan LK. et al. Genome-wide selective sweeps and gene-specific sweeps in natural bacterial populations. ISME J. 2016;10:1589–601. 26744812 10.1038/ismej.2015.241 PMC4918448

[bib4] Bokulich NA, Kaehler BD, Rideout JR. et al. Optimizing taxonomic classification of marker-gene amplicon sequences with QIIME 2’s q2-feature-classifier plugin. Microbiome. 2018;6:1–17. 29773078 10.1186/s40168-018-0470-z PMC5956843

[bib5] Bolyen E, Rideout JR, Dillon MR. et al. Reproducible, interactive, scalable and extensible microbiome data science using QIIME 2. Nat Biotechnol. 2019;37:852–7. 31341288 10.1038/s41587-019-0209-9 PMC7015180

[bib6] Bruneel O, Duran R, Casiot C. et al. Diversity of microorganisms in Fe-As-rich acid mine drainage waters of Carnoulès, France. Appl Environ Microb. 2006;72:551–6. 10.1128/AEM.72.1.551-556.2006 PMC1352176 16391091

[bib7] Callahan BJ, McMurdie PJ, Rosen MJ. et al. DADA2: high-resolution sample inference from Illumina amplicon data. Nat Methods. 2016;13:581–3. 27214047 10.1038/nmeth.3869 PMC4927377

[bib8] Chan CS, Dykes GE, Hoover RL. et al. Gallionellaceae in rice root plaque: metabolic roles in iron oxidation, nutrient cycling, and plant interactions. Appl Environ Microb. 2023;89:1–23. 10.1128/aem.00570-23 PMC10734482 38009924

[bib9] Chan CS, Fakra SC, Edwards DC. et al. Iron oxyhydroxide mineralization on microbial extracellular polysaccharides. Geochim Cosmochim Acta. 2009;73:3807–18.

[bib10] Chaumeil PA, Mussig AJ, Hugenholtz P. et al. GTDB-Tk v2: memory friendly classification with the genome taxonomy database. Bioinformatics. 2022;38:5315–6. 36218463 10.1093/bioinformatics/btac672 PMC9710552

[bib11] Chen S. Ultrafast one-pass FASTQ data preprocessing, quality control, and deduplication using fastp. Imeta. 2023;2:1–5. 10.1002/imt2.107 PMC10989850 38868435

[bib12] Chiu BK, Kato S, Mcallister SM. et al. Novel pelagic iron-oxidizing zetaproteobacteria from the Chesapeake Bay oxic-anoxic transition zone. Appl Environ Microb. 2017;8:1–16. 10.3389/fmicb.2017.01280 PMC5513912 28769885

[bib13] Cooper RE, Wegner C-E, McAllister SM. et al. Draft genome sequence of Sideroxydans sp. Strain CL21, an Fe(II)-oxidizing bacterium. Microbiol Resour Announc. 2020;9:10.1128/mra.01444-19. PMC6952672 31919186

[bib14] Cornell R, Schwertmann U.“Thermodynamics of the Fe-O_2_-H_2_O system.” In The Iron Oxides. Weinheim: Wiley-VCH Verlag GmbH& Co. KGaA, 2003, 185–99.

[bib15] Druschel GK, Emerson D, Sutka R. et al. Low-oxygen and chemical kinetic constraints on the geochemical niche of neutrophilic iron(II) oxidizing microorganisms. Geochim Cosmochim Acta. 2008;72:3358–70.

[bib16] Emerson D, Field EK, Chertkov O. et al. Comparative genomics of freshwater Fe-oxidizing bacteria: implications for physiology, ecology, and systematics. Front Microbiol. 2013;4:1–17. 24062729 10.3389/fmicb.2013.00254 PMC3770913

[bib17] Emerson D, Fleming EJ, McBeth JM. Iron-oxidizing bacteria: an environmental and genomic perspective. Annu Rev Microbiol. 2010;64:561–83. 20565252 10.1146/annurev.micro.112408.134208

[bib18] Emerson D, Floyd MM. Enrichment and isolation of iron-oxidizing bacteria at neutral pH. Methods Enzymol. 2005;397:112–23. 16260287 10.1016/S0076-6879(05)97006-7

[bib19] Emerson D, Moyer C. Isolation and characterization of novel iron-oxidizing bacteria that grow at circumneutral pH. Appl Environ Microb. 1997;63:4784–92. 10.1128/aem.63.12.4784-4792.1997 PMC168801 9406396

[bib20] Emerson D, Revsbech NP. Investigation of an iron-oxidizing microbial mat community located near Aarhus, Denmark: field studies. Appl Environ Microb. 1994;60:4022–31. 10.1128/aem.60.11.4022-4031.1994 PMC201931 16349433

[bib21] Emerson D, Weiss JV. Bacterial iron oxidation in circumneutral freshwater habitats: findings from the field and the laboratory. Geomicrobiol J. 2004;21:405–14.

[bib22] Fabisch M, Beulig F, Akob DM. et al. Surprising abundance of Gallionella-related iron oxidizers in creek sediments at pH 4.4 or at high heavy metal concentrations. Front Microbiol. 2013;4:1–12. 24385973 10.3389/fmicb.2013.00390 PMC3866512

[bib23] Ferris FG, Hallberg RO, Lyvén B. et al. Retention of strontium, cesium, lead and uranium by bacterial iron oxides from a subterranean environment. Appl Geochem. 2000;15:1035–42.

[bib24] Field HR, Whitaker AH, Henson JA. et al. Sorption of copper and phosphate to diverse biogenic iron (oxyhydr)oxide deposits. Sci Total Environ. 2019;697:134111. 31487593 10.1016/j.scitotenv.2019.134111

[bib25] Fortin D, Langley S. Formation and occurrence of biogenic iron-rich minerals. Earth Sci Rev. 2005;72:1–19.

[bib26] Garber AI, Nealson KH, Okamoto A. et al. FeGenie: a comprehensive tool for the identification of iron genes and iron gene neighborhoods in genome and metagenome assemblies. Front Microbiol. 2020;11:1–23. 32082281 10.3389/fmicb.2020.00037 PMC7005843

[bib27] Gupta SG, Agate AD. Preservation of thiobacillus ferroxidans and thiobacillus thiooxidans with activity check. Antonie Van Leeuwenhoek. 1986;52:121–7. 3524447 10.1007/BF00429315

[bib28] Hanert HH. The genus Gallionella. In The Prokaryotes, Starr M., Stolp H., Truper H., Balows A., Schlegel H. (eds). Berlin: Springer, 1981, 509–15.

[bib29] Hanert HH. The Genus Gallionella BT—The Prokaryotes: Proteobacteria: Delta, Epsilon Subclass. In: Dworkin M, Falkow S, Rosenberg E, et al. (eds.). New York, NY: Springer New York, 2006,990–5.

[bib30] Hannon GJ. FASTX-Toolkit. http://hannonlab.cshl.edu/fastx_toolkit. 2010.

[bib31] Hassan Z, Sultana M, Westerhoff HV. et al. Iron cycling potentials of arsenic contaminated groundwater in Bangladesh as revealed by enrichment cultivation. Geomicrobiol J. 2016;33:779–92.

[bib32] He S, Tominski C, Kappler A. et al. Metagenomic analyses of the autotrophic Fe(II)-oxidizing, nitrate-reducing enrichment culture KS. Appl Environ Microb. 2016;82:2656–68. 10.1128/AEM.03493-15 PMC4836422 26896135

[bib33] Herlemann DPR, Labrenz M, Jürgens K. et al. Transitions in bacterial communities along the 2000 km salinity gradient of the Baltic Sea. ISME J. 2011;5:1571–9. 21472016 10.1038/ismej.2011.41 PMC3176514

[bib34] Hoover RL, Keffer JL, Polson SW. et al. Gallionellaceae pangenomic analysis reveals insight into phylogeny, metabolic flexibility, and iron oxidation mechanisms. Appl Environ Microb. 2023;8:1–28. 10.1128/msystems.00038-23 PMC10734462 37882557

[bib35] Jakus N, Blackwell N, Straub D. et al. Presence of Fe(II) and nitrate shapes aquifer-originating communities leading to an autotrophic enrichment dominated by an Fe(II)-oxidizing Gallionellaceae sp. FEMS Microbiol Ecol. 2021;97:1–14. 10.1093/femsec/fiab145 34724047

[bib36] Joshi NA, Fass JN. Sickle: A sliding-window, adaptive, quality-based trimming tool for FastQ files (Version 1.33). 2011. https://github.com/najoshi/sickle.

[bib37] Kadnikov VV, Ivasenko DA, Beletskii AV. et al. A novel uncultured bacterium of the family Gallionellaceae: description and genome reconstruction based on metagenomic analysis of microbial community in acid mine drainage. Microbiology. 2016;85:449–61. 28853774

[bib38] Kanehisa M, Furumichi M, Tanabe M. et al. KEGG: new perspectives on genomes, pathways, diseases and drugs. Nucleic Acids Res. 2017;45:D353–61. 27899662 10.1093/nar/gkw1092 PMC5210567

[bib39] Kanehisa M, Sato Y, Morishima K. BlastKOALA and GhostKOALA: KEGG tools for functional characterization of genome and metagenome sequences. J Mol Biol. 2016;428:726–31. 26585406 10.1016/j.jmb.2015.11.006

[bib40] Kato S, Chan C, Itoh T. et al. Functional gene analysis of freshwater iron-rich flocs at circumneutral pH and isolation of a stalk-forming microaerophilic iron-oxidizing bacterium. Appl Environ Microb. 2013;79:5283–90. 10.1128/AEM.03840-12 PMC3753938 23811518

[bib41] Kato S, Itoh T, Iino T. et al. Sideroxyarcus emersonii gen. nov. Sp. nov., a neutrophilic, microaerobic iron- and thiosulfate-oxidizing bacterium isolated from iron-rich wetland sediment. Int J Syst Evol Microbiol. 2022;72, 005347. 10.1099/ijsem.0.005347 35476601

[bib42] Kato S, Krepski S, Chan C. et al. Ferriphaselus amnicola gen. nov., sp. nov., a neutrophilic, stalk-forming, iron-oxidizing bacterium isolated from an iron-rich groundwater seep. Int J Syst Evol Microbiol. 2014;64:921–5. 24425821 10.1099/ijs.0.058487-0

[bib43] Katsoyiannis IA, Werner Althoff H, Bartel H. et al. The effect of groundwater composition on uranium(VI) sorption onto bacteriogenic iron oxides. Water Res. 2006;40:3646–52. 16908045 10.1016/j.watres.2006.06.032

[bib44] Katsoyiannis IA, Zouboulis AI. Application of biological processes for the removal of arsenic from groundwaters. Water Res. 2004;38:17–26. 14630099 10.1016/j.watres.2003.09.011

[bib45] Keuter S, Koch H, Sass K. et al. Some like it cold: the cellular organization and physiological limits of cold-tolerant nitrite-oxidizing Nitrotoga. Environ Microbiol. 2022;24:2059–77. 35229435 10.1111/1462-2920.15958

[bib46] Khalifa A, Nakasuji Y, Saka N. et al. Ferrigenium kumadai gen. Nov., sp. nov., a microaerophilic iron-oxidizing bacterium isolated from a paddy field soil. Int J Syst Evol Microbiol. 2018;68:2587–92. 29944111 10.1099/ijsem.0.002882

[bib47] Kikuchi S, Kashiwabara T, Shibuya T. et al. Molecular-scale insights into differences in the adsorption of cesium and selenium on biogenic and abiogenic ferrihydrite. Geochim Cosmochim Acta. 2019;251:1–14.

[bib48] Klappenbach JA, Dunbar JM, Schmidt TM. rRNA operon copy number reflects ecological strategies of bacteria. Appl Environ Microb. 2000;66:1328–33. 10.1128/aem.66.4.1328-1333.2000 PMC91988 10742207

[bib49] Konstantinidis KT, Tiedje JM. Genomic insights that advance the species definition for prokaryotes. Proc Natl Acad Sci USA. 2005;102:2567–72. 15701695 10.1073/pnas.0409727102 PMC549018

[bib50] Konstantinidis KT, Tiedje JM. Prokaryotic taxonomy and phylogeny in the genomic era: advancements and challenges ahead. Curr Opin Microbiol. 2007;10:504–9. 17923431 10.1016/j.mib.2007.08.006

[bib51] Krepski ST, Hanson TE, Chan CS. Isolation and characterization of a novel biomineral stalk-forming iron-oxidizing bacterium from a circumneutral groundwater seep. Environ Microbiol. 2012;14:1671–80. 22151253 10.1111/j.1462-2920.2011.02652.x

[bib52] Kucera S, Wolfe RS. A selective enrichment method for Gallionella Ferruginea. J Bacteriol. 1957;74:344–9. 13475247 10.1128/jb.74.3.344-349.1957 PMC314645

[bib53] Langmuir D. Aqueous Environmental Geochemistry. New Jersey: Prentice Hall. 1997. https://books.google.co.jp/books?id=RroPAQAAIAAJ

[bib54] Liljeqvist M, Ossandon FJ, González C. et al. Metagenomic analysis reveals adaptations to a cold-adapted lifestyle in a low-temperature acid mine drainage stream. FEMS Microbiol Ecol. 2015;91:1–12. 10.1093/femsec/fiv011 25764459

[bib55] Lüdecke C, Reiche M, Eusterhues K. et al. Acid-tolerant microaerophilic Fe(II)-oxidizing bacteria promote Fe(III)-accumulation in a fen. Environ Microbiol. 2010;12:2814–25. 20545739 10.1111/j.1462-2920.2010.02251.x

[bib56] Lueder U, Maisch M, Jørgensen BB. et al. Growth of microaerophilic Fe(II)-oxidizing bacteria using Fe(II) produced by Fe(III) photoreduction. Geobiology. 2022;20:421–34. 35014744 10.1111/gbi.12485

[bib57] Magoč T, Salzberg SL. FLASH: fast length adjustment of short reads to improve genome assemblies. Bioinformatics. 2011;27:2957–63. 21903629 10.1093/bioinformatics/btr507 PMC3198573

[bib58] Maisch M, Lueder U, Laufer K. et al. Contribution of microaerophilic iron(II)-oxidizers to iron(III) mineral formation. Environ Sci Technol. 2019;53:8197–204. 31203607 10.1021/acs.est.9b01531

[bib59] Maldonado R, Jimenez J, Casadesus J. Changes of ploidy during the Azotobacter vinelandii growth cycle. J Bacteriol. 1994;176:3911–9. 8021173 10.1128/jb.176.13.3911-3919.1994 PMC205588

[bib60] Melton ED, Swanner ED, Behrens S. et al. The interplay of microbially mediated and abiotic reactions in the biogeochemical Fe cycle. Nat Rev Micro. 2014;12:797–808. 10.1038/nrmicro3347 25329406

[bib61] Miot J, Benzerara K, Obst M. et al. Extracellular iron biomineralization by photoautotrophic iron-oxidizing bacteria. Appl Environ Microb. 2009;75:5586–91. 10.1128/AEM.00490-09 PMC2737918 19592528

[bib62] Mitsunobu S, Hamanura N, Kataoka T. et al. Arsenic attenuation in geothermal streamwater coupled with biogenic arsenic(III) oxidation. Appl Geochem. 2013;35:154–60.

[bib63] Mitsunobu S, Shiraishi F, Makita H. et al. Bacteriogenic Fe(III) (Oxyhydr)oxides characterized by synchrotron microprobe coupled with spatially resolved phylogenetic analysis. Environ Sci Technol. 2012;46:3304–11. 22360427 10.1021/es203860m

[bib64] Naik A, Patel P. Isolation and Identification of Iron-oxidizing Microbes BT—Practical Handbook on Agricultural Microbiology. In Amaresan N., Patel P., Amin D. (eds). New York, NY: Springer US, 2022, 223–30.

[bib65] Naruse T, Ban Y, Yoshida T. et al. Community structure of microaerophilic iron-oxidizing bacteria in Japanese paddy field soils. Soil Sci Plant Nutr. 2019;65:460–70.

[bib66] Neubauer SC, Emerson D, Megonigal JP. Life at the energetic edge: kinetics of circumneutral iron oxidation by lithotrophic iron-oxidizing bacteria isolated from the wetland-plant rhizosphere. Appl Environ Microb. 2002;68:3988–95. 10.1128/AEM.68.8.3988-3995.2002 PMC124031 12147500

[bib67] Parkhurst DL, Appelo CAJ. Description of input and examples for PHREEQC version 3—a computer program for speciation, batch-reaction, one-dimensional transport, and inverse geochemical calculations: U.S. Geo Survey Techniq Methods. Colorado: 2013, 497.

[bib68] Parks DH, Chuvochina M, Rinke C. et al. GTDB: an ongoing census of bacterial and archaeal diversity through a phylogenetically consistent, rank normalized and complete genome-based taxonomy. Nucleic Acids Res. 2022;50:D785–94. 34520557 10.1093/nar/gkab776 PMC8728215

[bib69] Parks DH, Rinke C, Chuvochina M. et al. Recovery of nearly 8000 metagenome-assembled genomes substantially expands the tree of life. Nat Microbiol. 2017;2:1533–42. 28894102 10.1038/s41564-017-0012-7

[bib70] Pecoraro V, Zerulla K, Lange C. et al. Quantification of ploidy in proteobacteria revealed the existence of monoploid, (mero-)oligoploid and polyploid species. PLoS One. 2011;6:e16392. 21305010 10.1371/journal.pone.0016392 PMC3031548

[bib71] Quast C, Pruesse E, Yilmaz P. et al. The SILVA ribosomal RNA gene database project: improved data processing and web-based tools. Nucleic Acids Res. 2013;41:D590–6. 23193283 10.1093/nar/gks1219 PMC3531112

[bib72] Roden EE, Sobolev D, Glazer B. et al. Potential for microscale bacterial Fe redox cycling at the aerobic-Anaerobic interface. Geomicrobiol J. 2004;21:379–91.

[bib73] Rodriguez-R LM, Konstantinidis KT. The enveomics collection: a toolbox for specialized analyses of microbial genomes and metagenomes. PeerJ Preprints. 2016;4:e1900v1.

[bib74] Saywell LG, Cunningham BB. Determination of iron: colorimetric o-phenanthroline method. Ind Eng Chem Anal Ed. 1937;9:67–9.

[bib75] Scheiner D. Determination of ammonia and kjeldahl nitrogen by indophenol method. Water Res. 1976;10:31–6.

[bib76] Shiraishi F, Matsumura Y, Chihara R. et al. Depositional processes of microbially colonized manganese crusts, Sambe hot spring, Japan. Geochim Cosmochim Acta. 2019;258:1–18.

[bib77] Singer PC, Stumm W. Acid mine drainage: the rate-determining step. Science. 1970;167:1121–3. 17829406 10.1126/science.167.3921.1121

[bib78] Sowers TD, Harrington JM, Polizzotto ML. et al. Sorption of arsenic to biogenic iron (oxyhydr)oxides produced in circumneutral environments. Geochim Cosmochim Acta. 2017;198:194–207.

[bib79] Stackebrandt E, Ebers J. Taxonomic parameters revisited: tarnished gold standards. Microbiology Today. 2006;33:152–5.

[bib80] Stumm W, Morgan JJ. Aquatic Chemistry: chemical Equilibria and Rates in Natural Waters. New York: Wiley, 2012.

[bib81] Summers ZM, Gralnick JA, Bond DR. Cultivation of an obligate Fe(II)-oxidizing lithoautotrophic bacterium using electrodes. mBio. 2013;4:1–5. 10.1128/mBio.00420-12 PMC3560526 23362318

[bib82] Suzuki MT, Taylor LT, DeLong EF. Quantitative analysis of small-subunit rRNA genes in mixed microbial populations via 5′-nuclease assays. Appl Environ Microb. 2000;66:4605–14. 10.1128/aem.66.11.4605-4614.2000 PMC92356 11055900

[bib83] Tanizawa Y, Fujisawa T, Nakamura Y. DFAST: a flexible prokaryotic genome annotation pipeline for faster genome publication. Bioinformatics. 2018;34:1037–9. 29106469 10.1093/bioinformatics/btx713 PMC5860143

[bib84] Tokuyama T. A method for long-term preservation of a chemoautotrophic ammonia-oxidizing bacterium, nitrosomonas using silica gel powder. Bull Japanese Soc Microb Ecol. 1994;9:119–23.

[bib85] Tong H, Liu C, Hao L. et al. Biological Fe(II) and As(III) oxidation immobilizes arsenic in micro-oxic environments. Geochim Cosmochim Acta. 2019;265:96–108.

[bib86] Tong H, Zheng C, Li B. et al. Microaerophilic oxidation of Fe(II) coupled with simultaneous carbon fixation and As(III) oxidation and sequestration in karstic paddy soil. Environ Sci Technol. 2021;55:3634–44. 33411520 10.1021/acs.est.0c05791

[bib87] Wang J, Muyzer G, Bodelier PLE. et al. Diversity of iron oxidizers in wetland soils revealed by novel 16S rRNA primers targeting Gallionella-related bacteria. ISME J. 2009;3:715–25. 19225553 10.1038/ismej.2009.7

[bib88] Watanabe T, Khalifa A, Asakawa S. Complete genome sequence of ferrigenium kumadai An22, a microaerophilic iron-oxidizing bacterium isolated from a Paddy Field soil. Microbiol Resour Announc. 2021;10:1–2. 10.1128/MRA.00346-21 PMC8265223 34236217

[bib89] Weiss JV, Rentz JA, Plaia T. et al. Characterization of neutrophilic Fe(II)-oxidizing bacteria isolated from the rhizosphere of wetland plants and description of Ferritrophicum radicicola gen. nov. Sp. nov., and sideroxydans paludicola sp. nov. Geomicrobiol J. 2007;24:559–70.

[bib90] White DE. Magmatic, connate, and metamorphic waters. Geol Soc America Bull. 1957;68:1659–82.

[bib91] Zhou N, Keffler JL, Polson SW. et al. Unraveling Fe(II)-oxidizing mechanisms in a facultative Fe(II) oxidizer, sideroxydans lithotrophicus strain ES-1, via culturing, transcriptomics, and Reverse Transcription-quantitative PCR. Appl Environ Microb. 2022;88:1–16. 10.1128/AEM.01595-21 PMC8788666 34788064

