# Supplementary material for: Custom-made medium approach for effective enrichment and isolation of chemolithotrophic iron-oxidizing bacteria
Source: FEMS Microbiol Ecol. 2025 May 5;101(6):fiaf051. doi: 10.1093/femsec/fiaf051 (PMC12089753; doi:10.1093/femsec/fiaf051)
Supplement: fiaf051_Supplemental_File [file fiaf051_supplemental_file.pdf]

Supplementary Material for

Custom-made medium approach for effective enrichment and isolation of chemolithotrophic iron-oxidizing bacteria

T. Uchijima[1], S. Kato[2], K. Tanimoto[1], F. Shiraishi[3], N. Hamamura[4], K. Tokunaga[5], H. Makita[6,7], M. Ohkuma[2], S. Mitsunobu[1]*

[1]Department of Science and Technology for Biological Resources and Environment, Graduate School of Agriculture, Ehime University, 3-5-7 Tarumi, Matsuyama, Ehime 790-8566, Japan, [2]Japan Collection of Microorganisms, RIKEN BioResource Research Center, 3-1-1 Koyadai, Tsukuba, Ibaraki 305-0074, Japan, Earth and Planetary Systems Science Program, Graduate School of Advanced Science and Engineering, Hiroshima University, 1-3-1 Kagamiyama, Higashi-Hiroshima, Hiroshima 739-8526, Japan, [4]Department of Biology, Graduate School of Science, Kyushu University, 744 Motooka, Nishi-ku, Fukuoka 819-0395, Japan, [5]Ningyo-Toge Environmental Engineering Center, Japan Atomic Energy Agency, Okayama 708-0698, Japan, [6]Department of Ocean Sciences, Tokyo University of Marine Science and Technology, 4-5-7 Konan, Minato-ku, Tokyo 108-8477, Japan, [7]Institute for Extra-Cutting-Edge Science and Technology Avant-Garde Research, (X-star), Super-cutting-edge Grand and Advanced Research (SUGAR) Program, Japan Agency for Marine-Earth Science and Technology (JAMSTEC), Yokosuka, 237-0061, Japan

*Corresponding author: S. Mitsunobu, E-mail: mitsunobu.satoshi.dy@ehime-u.ac.jp

This PDF file includes:

Tables S1-S3

Figures S1-S5

Table S1. Primer sets used in the present study and PCR conditions.

	Target	Gene	Foward and Reverse primers	Sequence (5'–3')	Annealing temp. (°C)	PCR cycles	PCR product length (bp)	References
For amplicon sequencing	Eubacterial 16S rRNA	16S rRNA	341f	CCT ACG GGN GGC WGC AG	55	25-35	530	Herlemann et al. (2011)
(1st PCR)			805r	GAC TAC HVG GGT ATC TAA TCC				
For qPCR	Gallionellaceae 16S rRNA	16S rRNA	122F	TAT CGG AAC RTR TCC GGA	55	40	578	Naruse et al. (2019)
			Beta3R	ACG CAT TTC ACT GCT ACA CG				Ashelfold et al. (2002)
	Eubacterial 16S rRNA	16S rRNA	Bact-1369F	CGG TGA ATA CGT TCY CGG	56	40	142	Suzuki et al. (2000)
			Prok-1492R	GGW TAC CTT GTT ACG ACTT				

Table S2. Summary of selected functional genes in strains UT4 and UT5 based on the draft genome analyses.

The pink-colored cells are genes whose possessions were confirmed by their genome analyses.

<table>
<tr><th>Energy function</th><th>gene(s)</th><th>UT4</th><th>UT5</th></tr>
<tr><td rowspan="2">Fe oxidation</td><td>*cyc2*</td><td style="background:#fde4d8"></td><td style="background:#fde4d8"></td></tr>
<tr><td>*mtoAB*</td><td></td><td></td></tr>
<tr><td rowspan="2">O_2 reduction</td><td>*ccoN* (cbb_3)</td><td style="background:#fde4d8"></td><td style="background:#fde4d8"></td></tr>
<tr><td>*coxAB* (aa_3)</td><td></td><td></td></tr>
<tr><td rowspan="3">denitrification</td><td>*nirK*</td><td style="background:#fde4d8"></td><td style="background:#fde4d8"></td></tr>
<tr><td>*nirS*</td><td></td><td style="background:#fde4d8"></td></tr>
<tr><td>*norBC*</td><td></td><td style="background:#fde4d8"></td></tr>
<tr><td rowspan="3">S oxidation/reduction</td><td>*soxABXYZ*</td><td></td><td></td></tr>
<tr><td>*dsrAB/aprAB/sat*</td><td style="background:#fde4d8"></td><td style="background:#fde4d8"></td></tr>
<tr><td>*sqr*</td><td style="background:#fde4d8"></td><td style="background:#fde4d8"></td></tr>
<tr><td>fermentation or N fix</td><td>*nifJ*</td><td style="background:#fde4d8"></td><td style="background:#fde4d8"></td></tr>
<tr><td>fermentation</td><td>*ackA*</td><td style="background:#fde4d8"></td><td style="background:#fde4d8"></td></tr>
<tr><td rowspan="3">electron transport and ATP synthesis</td><td>*nuo*</td><td rowspan="3" style="background:#fde4d8"></td><td rowspan="3" style="background:#fde4d8"></td></tr>
<tr><td>*sdh*</td></tr>
<tr><td>ATPase</td></tr>
<tr><td>Rnf complex</td><td>*rnf*</td><td style="background:#fde4d8"></td><td style="background:#fde4d8"></td></tr>
</table>

<table>
<tr><th>Nutrient function</th><th>gene(s)</th><th>UT4</th><th>UT5</th></tr>
<tr><td>C fixation</td><td>*rbcL*</td><td rowspan="3" style="background:#fde4d8"></td><td rowspan="3" style="background:#fde4d8"></td></tr>
<tr><td>C storage (glycogen)</td><td>*glgABCP*</td></tr>
<tr><td>polysacch. hydrolysis</td><td>various</td></tr>
<tr><td>N_2 fixation</td><td>*nifHDK*</td><td rowspan="2"></td><td rowspan="2"></td></tr>
<tr><td>N storage</td><td>*cphA*</td></tr>
<tr><td>P storage (polyP)</td><td>*ppk, ppa, ppx*</td><td rowspan="2" style="background:#fde4d8"></td><td rowspan="2" style="background:#fde4d8"></td></tr>
<tr><td>P uptake</td><td>*pstB*</td></tr>
</table>

Table S3. Water chemistry in the environments Site A (groundwater) and Site B (hot spring) and the chemical compositions in a conventional MWMM and customized media for Sites A and B.

	**Sampling site / Medium type**	**Na (mM)**	**Mg (mM)**	**K (mM)**	**Ca (mM)**	**Fe(II) (μM)**	**NH_4 (μM)**	**PO_4 (μM)**	**Cl (mM)**	**SO_4 (mM)**	**DO (μM)**	**DIC (mM)**	**pH**	**Temperature (°C)**
**Water sample**	Site A	155	3.6	5.3	8.0	53	3.1	112	165	4.3	75	61	6.5	24
	Site B	56	3.1	3.7	6.1	80	1.7	3.0	93	2.5	36	31	6.1	30
**Medium**	**Customized medium**													
	for Site A	156	3.6	5.3	8.0	*	56	115	116	3.6	*	61	6.6	25
	for Site B	56	3.1	3.6	6.1	*	98	344	38	3.1	*	31	6.1	30
	**MWMM** (Emerson and Moyer, 1997)	5.0	0.81	0.57	0.68	*	18700	290	20	0.81	*	5.0	6.2-6.4	21

*The concentrations of dissolved Fe and O_2 are not stated because they vary with the medium depth in a tube as shown in Figure 4.

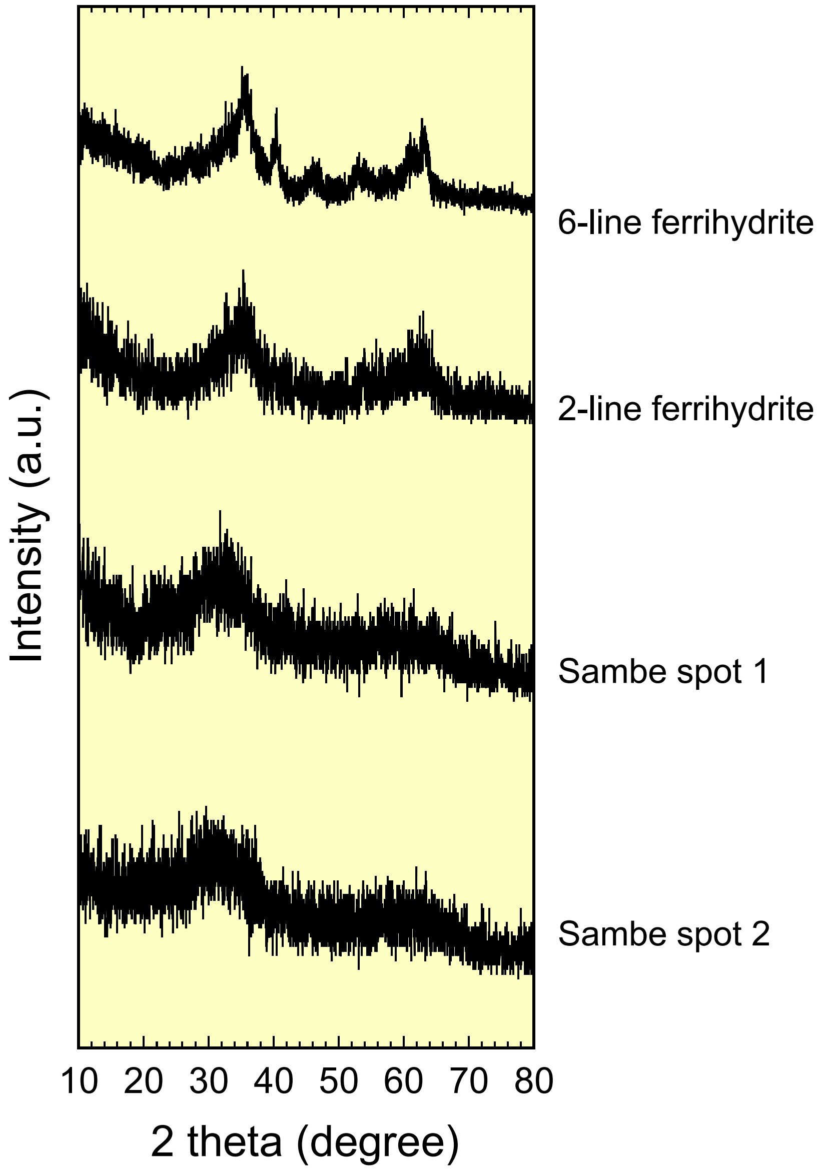

Fig. S1 XRD patterns of Sambe Fe precipitates and synthetic Fe(III) oxyhydroxides (2-line and 6-line ferrihydrite).

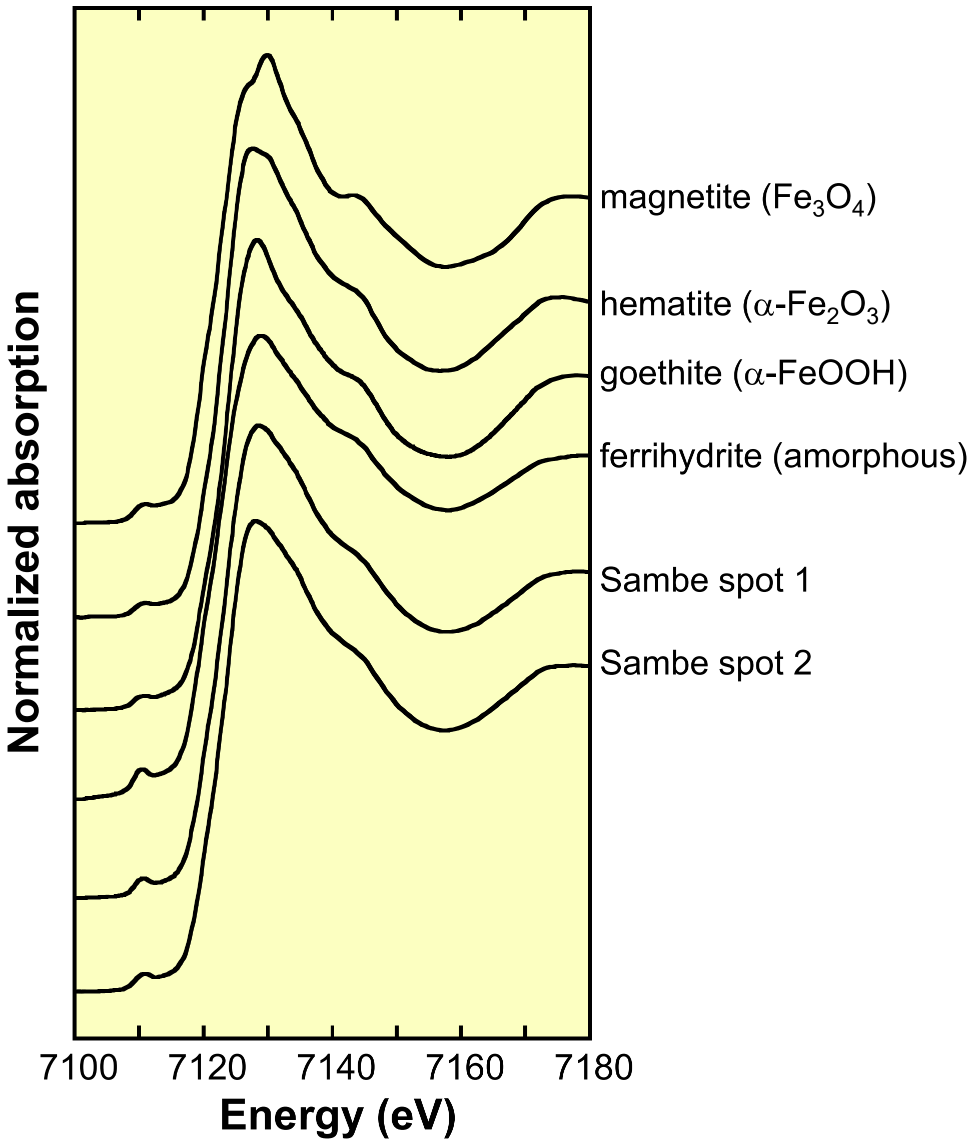

Fig. S2 Fe K-edge XANES spectra of Sambe Fe precipitates and standard Fe (hydr)oxides (magnetite, goethite, hematite, and 2-line ferrihydrite).

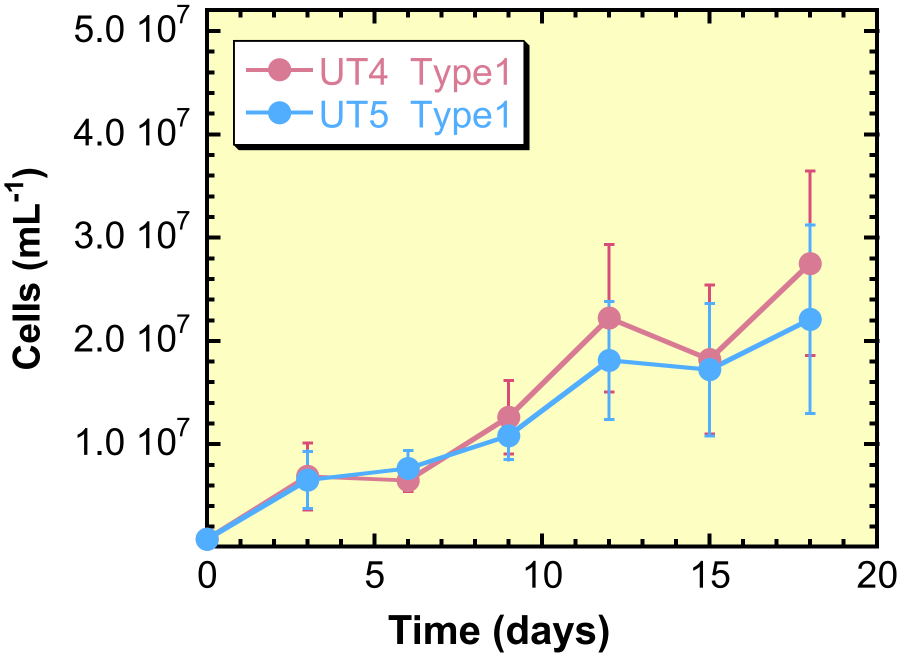

Fig. S3 Growth curves for strains UT4 and UT5 cultivated in medium Type 1 with FeS plug at 28°C. The doubling times stated in main text were calculated using data from 3 to 12 days.

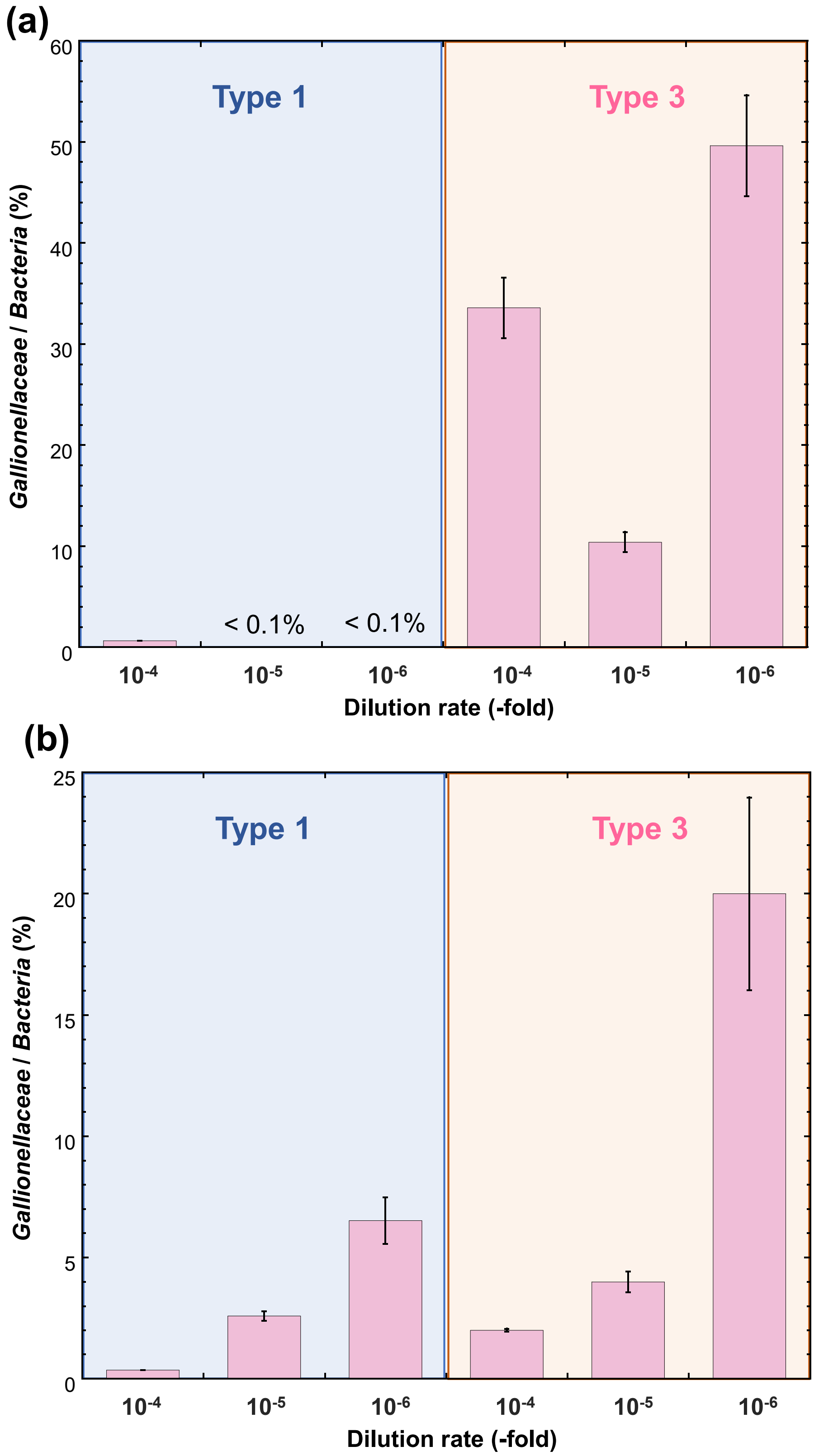

Fig. S4 *Gallionellaceae/Bacteria* ratios after the first enrichment with conventional Types 1 and our Type 3 media estimated by the qPCR with *Gallionellaceae* and *Bacteria* specific primers. The 10^{-4} to 10^{-6} diluted Fe-precipitates were inoculated to the media. The results in Site A (Fe-containing saline groundwater) and Site B (moderate temperature hot spring) were shown in figures (a) and (b), respectively.

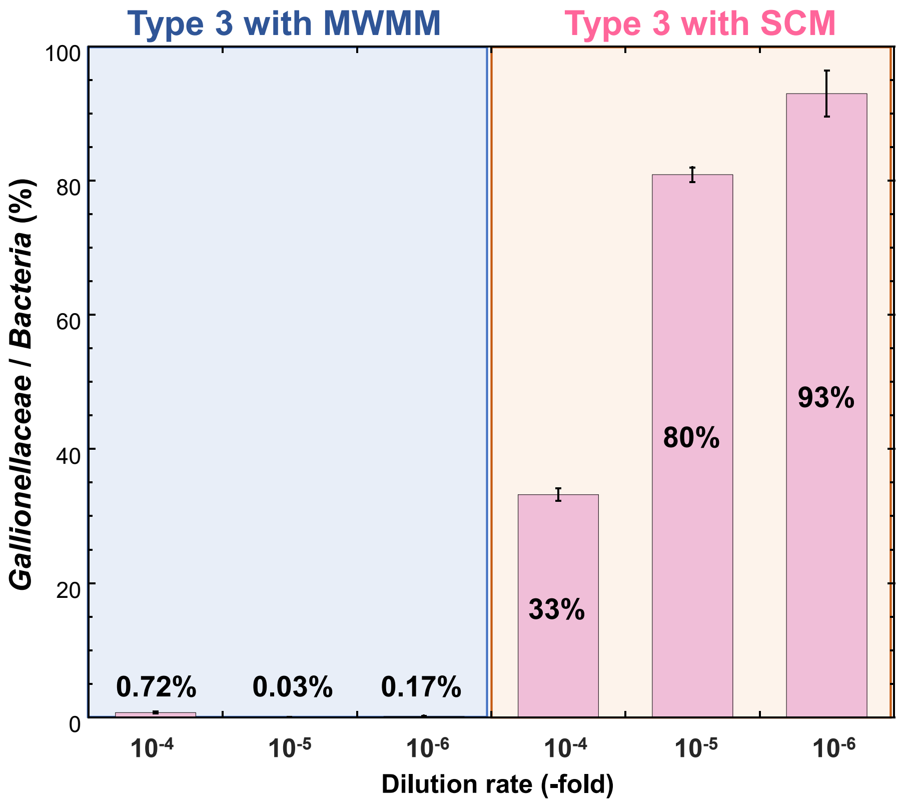

Fig. S5 *Gallionellaceae/Bacteria* ratios after the first enrichment using Type 3 with conventional MWMM and Sambe-custom medium (SCM) estimated by the qPCR with *Gallionellaceae* and *Bacteria* specific primers. The 10^{-4} to 10^{-6} diluted Fe-precipitates were inoculated to the media. The enrichment was conducted under the same conditions of temperature (28°C) and duration (14 days).
